# Supplementary material for: Pro-inflammatory macrophage activation does not require inhibition of oxidative phosphorylation
Source: EMBO Rep. 2025 Jan 3;26(4):982–1002. doi: 10.1038/s44319-024-00351-y (PMC11850891; doi:10.1038/s44319-024-00351-y)
Supplement: Supplementary file 1 — Appendix [file 44319_2024_351_MOESM1_ESM.pdf]

Appendix for

Pro-inflammatory macrophage activation does not require inhibition of oxidative phosphorylation

Andréa Ball *et al.*
* Corresponding author: E-mail: adivakaruni@mednet.ucla.edu

This PDF file includes:

Appendix Figure S1 – pg.2
Appendix Figure S2 – pg.3
Appendix Figure S3 – pg.4
Appendix Figure S4 – pg.5
Appendix Figure S5 – pg.6
Appendix Figure S6 – pg.7
Appendix Figure S7 – pg.8
Appendix Figure S8 – pg.9
Appendix Figure S9 – pg.10
Appendix Figure S10 – pg.11
Appendix Table S1 – pg.12-21

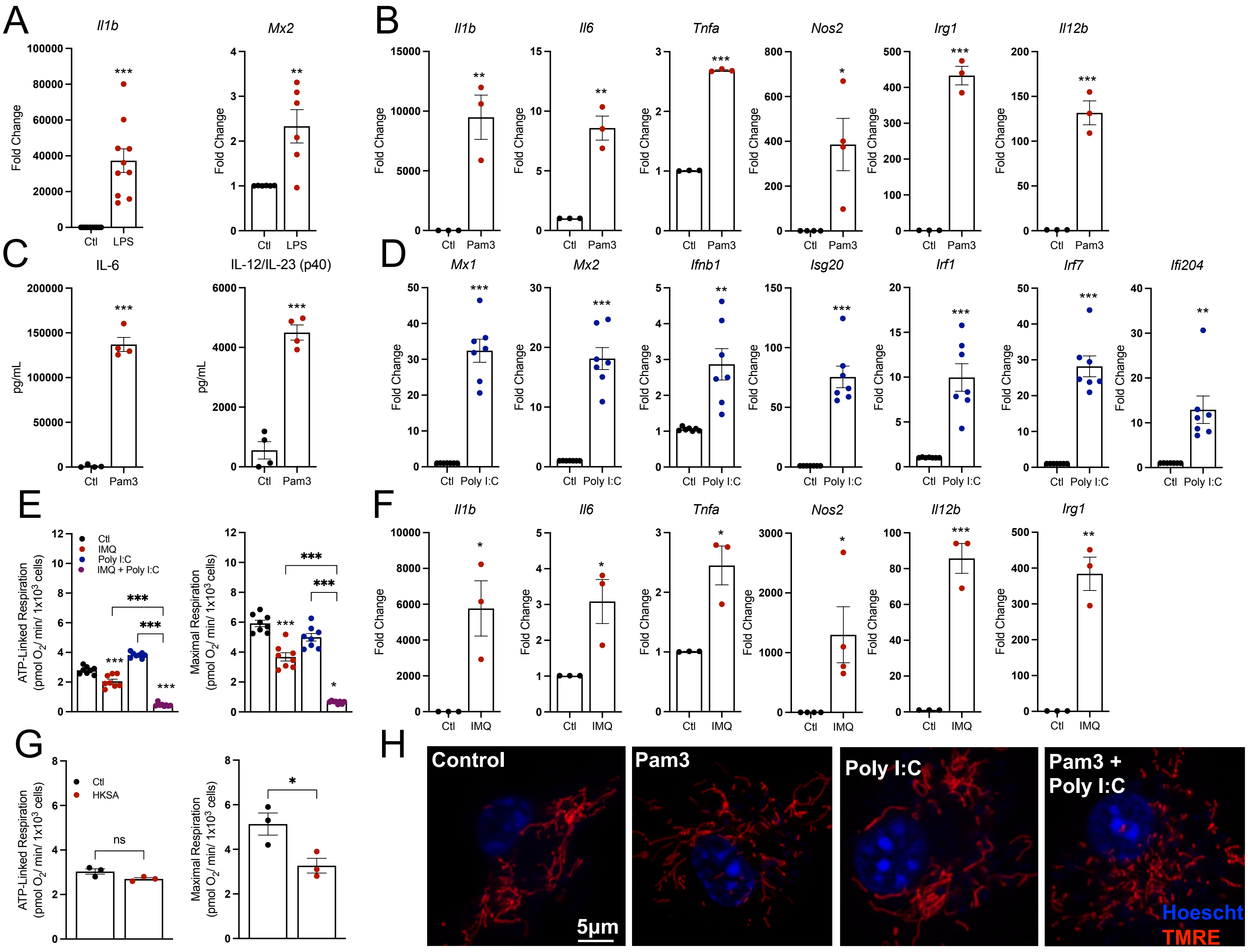

Appendix Figure S1: Induction of pro-inflammatory gene expression by multiple pro-inflammatory stimuli despite not all altering mitochondrial respiration. **A**) Pro-inflammatory gene expression of control (Ctl) and BMDMs treated with 50 ng/mL LPS for 24 hr. (n = 6-10). **B**) Pro-inflammatory gene expression for BMDMs treated with Pam3 for 24 hr. (n = 3-4). **C**) Cytokine levels in medium from control (Ctl) and BMDMs treated with Pam3 for 24 hr. (n = 4; values below the standard curve are denoted as zero). **D**) Pro-inflammatory gene expression of control (Ctl) and BMDMs treated with Poly I:C for 24 hr. (n = 7). **E**) ATP-linked and maximal respiration rates for BMDMs treated with Imiquimod (IMQ), Poly I:C, or IMQ + Poly I:C (n = 8). **F**) Pro-inflammatory gene expression for BMDMs treated with IMQ for 24 hr. (n = 3-4). **G**) ATP-linked respiration, maximal respiration, and lactate efflux rates for control (Ctl) and BMDMs treated with 10^7 heat-killed staphylococcus A (HKSA) for 24 hr. (n = 3). **H**) Representative images of mitochondrial morphology of BMDMs. Nuclei are stained with Hoechst and mitochondria are stained with TMRE. All data are mean ± SEM with statistical analysis conducted on data from biological replicates, each of which included multiple technical replicates, unless otherwise indicated. Statistical analysis for (**A-D**) and (**F-G**) was performed as an unpaired, two-tailed t-test**.** Statistical analysis for (**E**) and (**G**) was performed as an ordinary one-way, ANOVA followed by Tukey's *post hoc* multiple comparisons test.

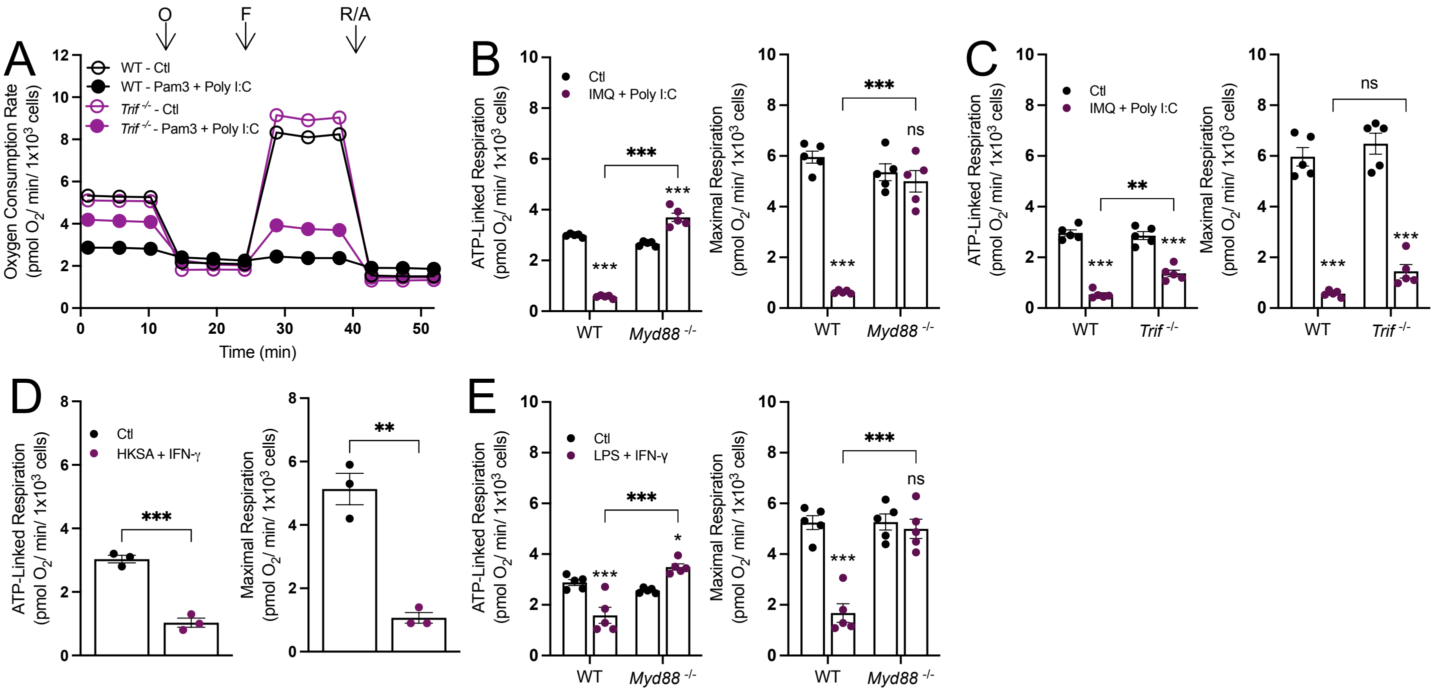

Appendix Figure S2: MyD88 and TRIF are required for multiple pro-inflammatory stimuli to decrease mitochondrial respiration. A) The oxygen consumption rates from a representative experiment for wildtype (WT) and TRIF-null (*Trif*$^{-/-}$) control (Ctl) BMDMs or BMDMs treated with Pam3 + Poly I:C for 24 hr. O, oligomycin; F, FCCP; R/A, rotenone/antimycin A (n = 1 biological with 5 technical replicates). **B**) ATP-linked and maximal respiration calculations for WT and *Myd88*$^{-/-}$ control (Ctl) and BMDMs treated with imiquimod (IMQ) + Poly I:C (n = 5). **C**) ATP-linked and maximal respiration calculations for WT and *Trif*$^{-/-}$ control (Ctl) and BMDMs treated with IMQ + Poly I:C (n = 5). **D**) ATP-linked and maximal respiration calculations for WT control (Ctl) and BMDMs treated with 10^7 heat-killed staphylococcus A (HKSA) + IFN-γ (n = 3). **E**) ATP-linked and maximal respiration calculations for WT and *Myd88*$^{-/-}$ control (Ctl) and BMDMs treated with LPS + IFN-γ (n = 5). All data are mean ± SEM with statistical analysis conducted on data from biological replicates, each of which included multiple technical replicates, unless otherwise indicated. Statistical analysis for (**B-E**) was performed as an ordinary two-way, ANOVA followed by Sídák's *post hoc* multiple comparisons test. Statistical analysis for (**F**) was performed as an unpaired, two-tailed t-test.

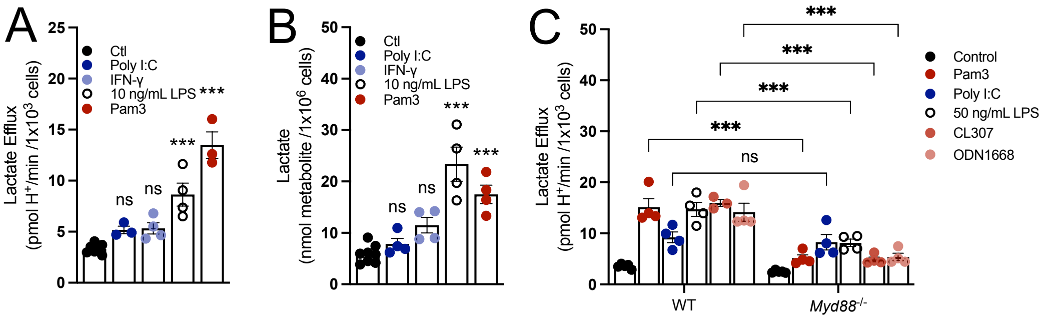

Appendix Figure S3: Increased glycolysis is dependent on MyD88-linked signaling. A) Lactate efflux rates for control (Ctl) BMDMs or BMDMs treated with Poly I:C, IFN-gamma, 10 ng/mL LPS, or Pam3 for 24 hr. (n = 3-4). **B**) Intracellular lactate abundance for Ctl BMDMs or BMDMs treated with Poly I:C, IFN-gamma, 10 ng/mL LPS, or Pam3 for 24 hr. (n = 4). **C**) Lactate efflux rates from wildtype (WT) and MyD88-null (*Myd88*$^{-/-}$) control (Ctl) BMDMs or BMDMs treated with Pam3, Poly I:C, 50 ng/mL LPS, CL307, or ODN1668 for 24 hr. (n = 3-4). All data are mean ± SEM with statistical analysis conducted on data from biological replicates, each of which included multiple technical replicates, unless otherwise indicated. Statistical analysis for (**A**) and (**B**) was performed as an ordinary one-way, ANOVA followed by Tukey's *post hoc* multiple comparisons test. Statistical analysis for (C) was performed as an ordinary two-way ANOVA followed by Sídák's *post hoc* multiple comparisons test.

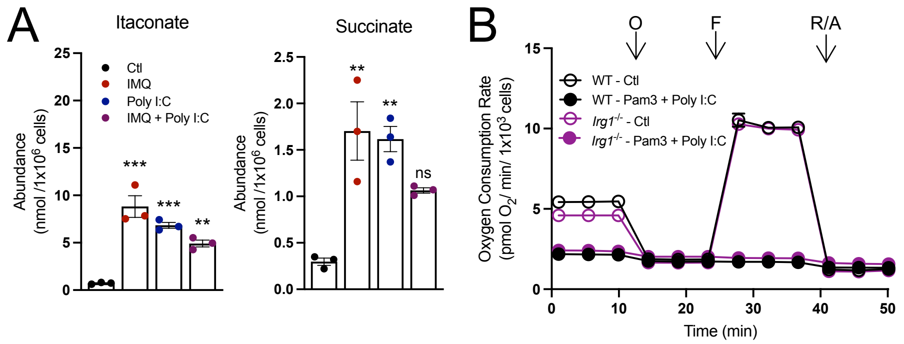

Appendix Figure S4: Multiple pro-inflammatory stimuli can accumulate itaconate and succinate independently of respiratory inhibition. **A**) Intracellular abundance of itaconate and succinate from control (Ctl) and BMDMs treated with IMQ, Poly I:C, or IMQ + Poly I:C (n = 3). **B**) The oxygen consumption rates from a representative experiment for wildtype (WT) and IRG1-null (*Irg1*$^{-/-}$) control (Ctl) and BMDMs activated with Pam3 + Poly I:C, O, oligomycin; F, carbonyl cyanide-p-trifluoromethoxyphenylhydrazone (FCCP); R/A, rotenone/antimycin A (n = 1 biological with 5 technical replicates). **C**) ATP-linked and maximal respiration rates for WT and *Irg1*$^{-/-}$ control (Ctl) and BMDMs treated with Pam3 + Poly I:C (n = 4). All data are mean ± SEM with statistical analysis conducted on data from biological replicates, each of which included multiple technical replicates, unless otherwise indicated. Statistical analysis for (**A**) was performed as an ordinary one-way, ANOVA followed by Tukey's *post hoc* multiple comparisons test. Statistical analysis for (**C**) was performed as an ordinary two-way, ANOVA followed by Sídák's *post hoc* multiple comparisons test.

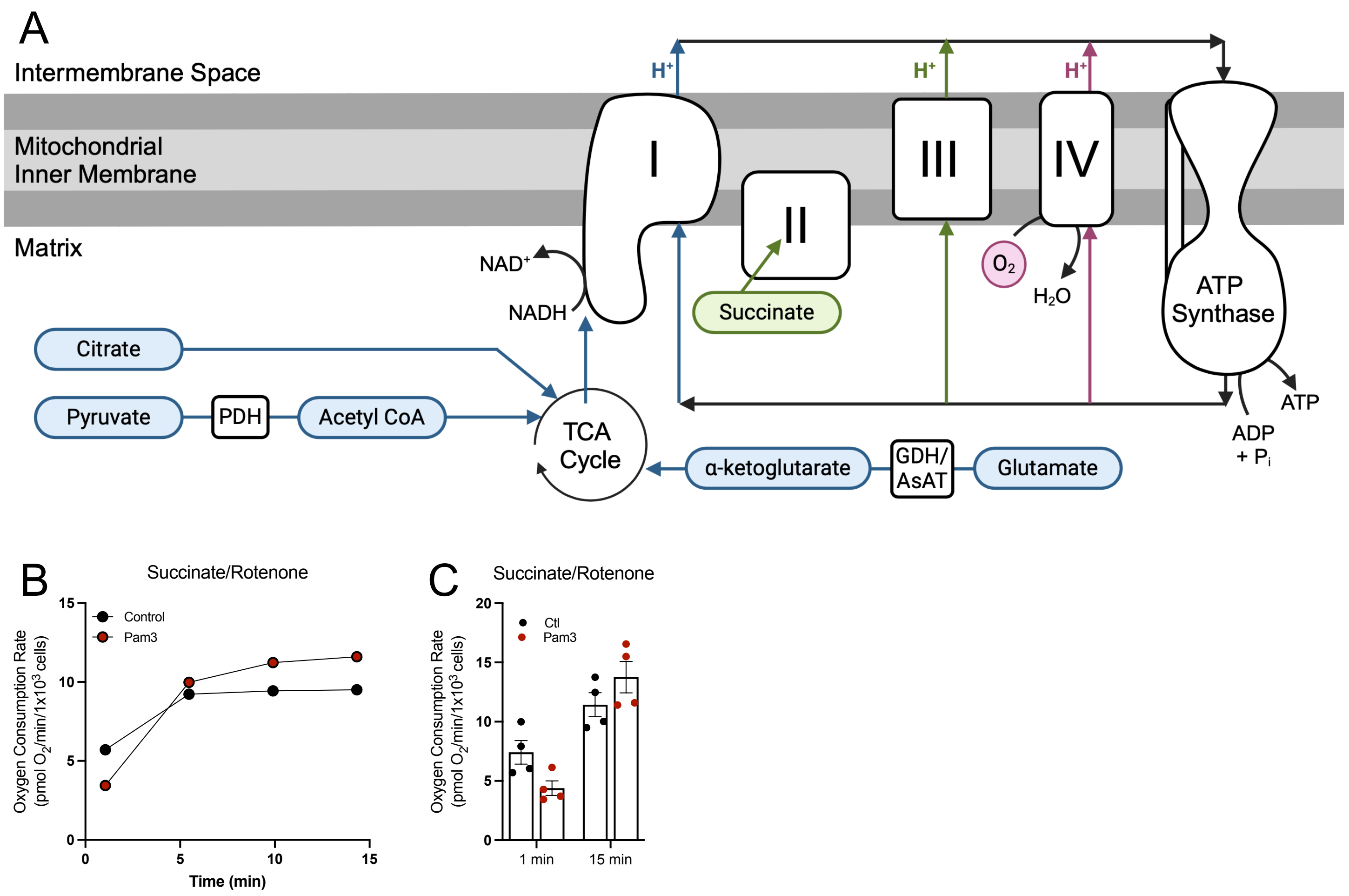

Appendix Figure S5: An overview of permeabilized respirometry and time dependent reversal of succinate dehydrogenase inhibition by itaconate. A) A graphical schematic of permeabilized respirometry substrates feeding into the TCA cycle or electron transport chain. PDH, pyruvate dehydrogenase; GDH, glutamate dehydrogenase; AsAT, aspartate aminotransferase. **B**) The oxygen consumption rates from a representative permeabilized respirometry assay with succinate and rotenone as the substrates and control (Ctl) and BMDMs activated with Pam3 for 24 hr. O, oligomycin; F, carbonyl cyanide-p-trifluoromethoxyphenylhydrazone (FCCP); R/A, rotenone/antimycin A (n = 1 biological with 5 technical replicates). **C**) Rates of oxygen consumption after the first and fourth measurements for control (Ctl) and BMDMs treated with Pam3 for 24 hr., permeabilized, and provided succinate and rotenone as substrates (n = 4).

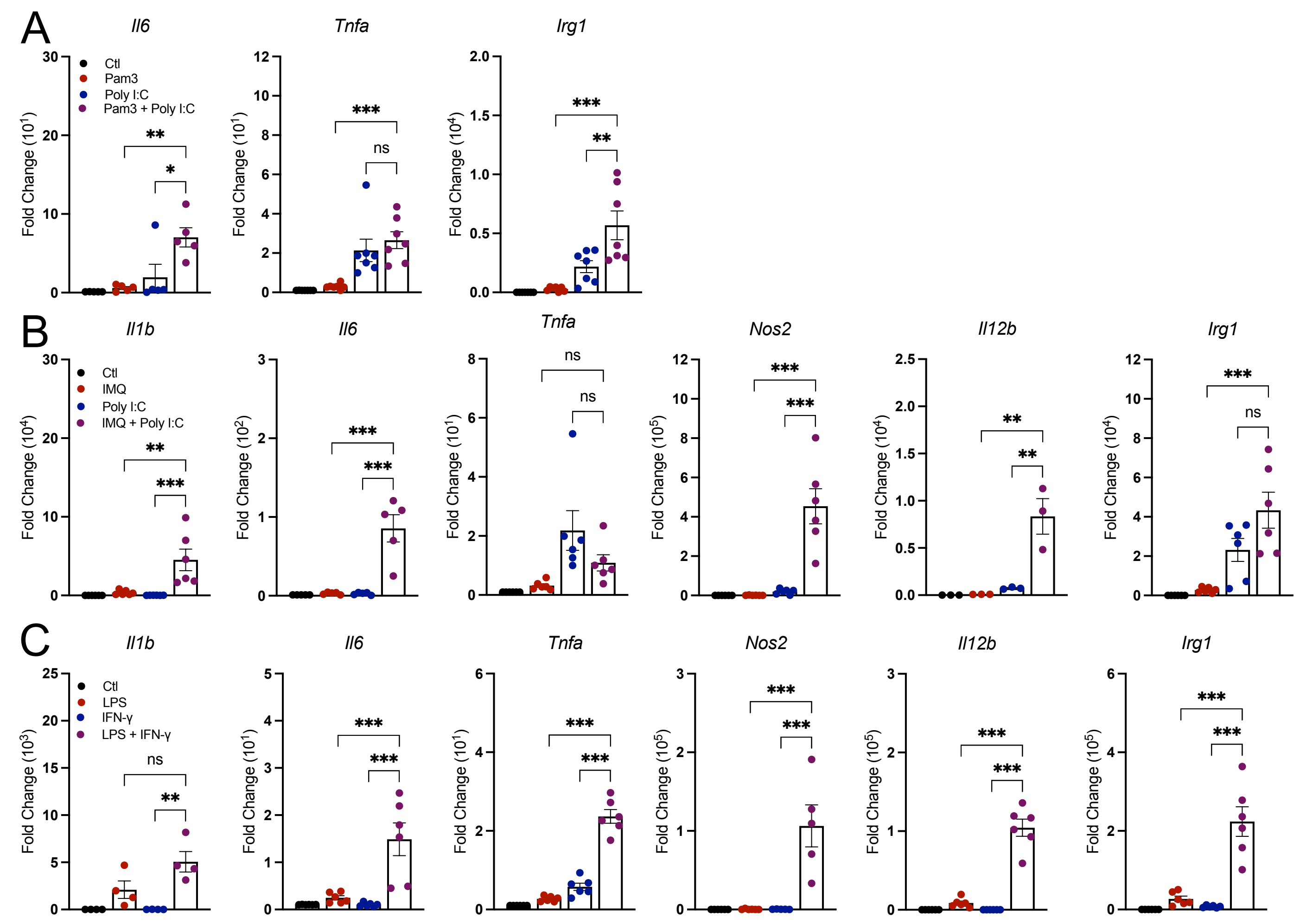

Appendix Figure S6: Synergistic increases in pro-inflammatory gene expression observed for multiple MyD88- and IFN-linked stimuli. A-C) Pro-inflammatory gene expression from BMDMs treated with the following groups: control (Ctl), Pam3, Poly I:C, or Pam3 + Poly I:C (n = 5-7) (**A**); control (Ctl), imiquimod (IMQ), Poly I:C, or IMQ + Poly I:C (n = 3-6) (**B**); or control (Ctl), 10 ng/mL LPS, IFN-γ, LPS + IFN-γ (n = 4-6) (**C**). All data are mean ± SEM with statistical analysis conducted on data from biological replicates, each of which included multiple technical replicates, unless otherwise indicated. Statistical analysis for (**A-C**) was performed as an ordinary one-way, ANOVA followed by Tukey's *post hoc* multiple comparisons test.

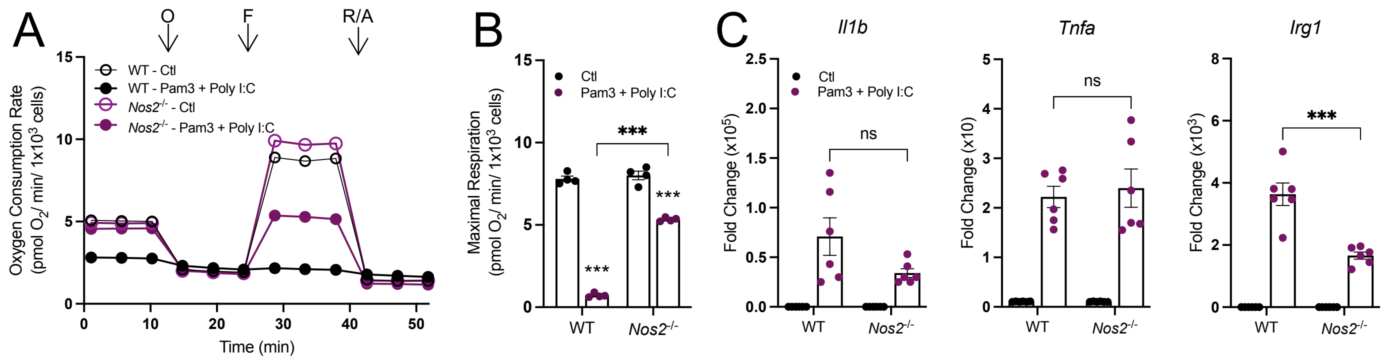

Appendix Figure S7: BMDMs from *Nos2*$^{-/-}$ mice have improved respiration but a comparable pro-inflammatory phenotype to WT BMDMs. A) The oxygen consumption rates from a representative experiment for wildtype (WT) or iNOS-null (*Nos2*$^{-/-}$) control (Ctl) and BMDMs treated with Pam3 + Poly I:C., O, oligomycin; F, carbonyl cyanide-p-trifluoromethoxyphenylhydrazone (FCCP); R/A, rotenone/antimycin A (n = 1 biological with 5 technical replicates). **B**) Maximal respiration rates for WT and *Nos2*$^{-/-}$ control (Ctl) and BMDMs treated with Pam3 + Poly I:C (n = 4). **C**) Pro-inflammatory gene expression relative to control for WT and *Nos2*$^{-/-}$ control (Ctl) and BMDMs treated with Pam3 + Poly I:C for 24 hr. (n = 6). All data are mean ± SEM with statistical analysis conducted on data from biological replicates, each of which included multiple technical replicates, unless otherwise indicated. Statistical analysis for (**B**) and (**C**) was performed as an ordinary two-way, ANOVA followed by Tukey's *post hoc* multiple comparisons test.

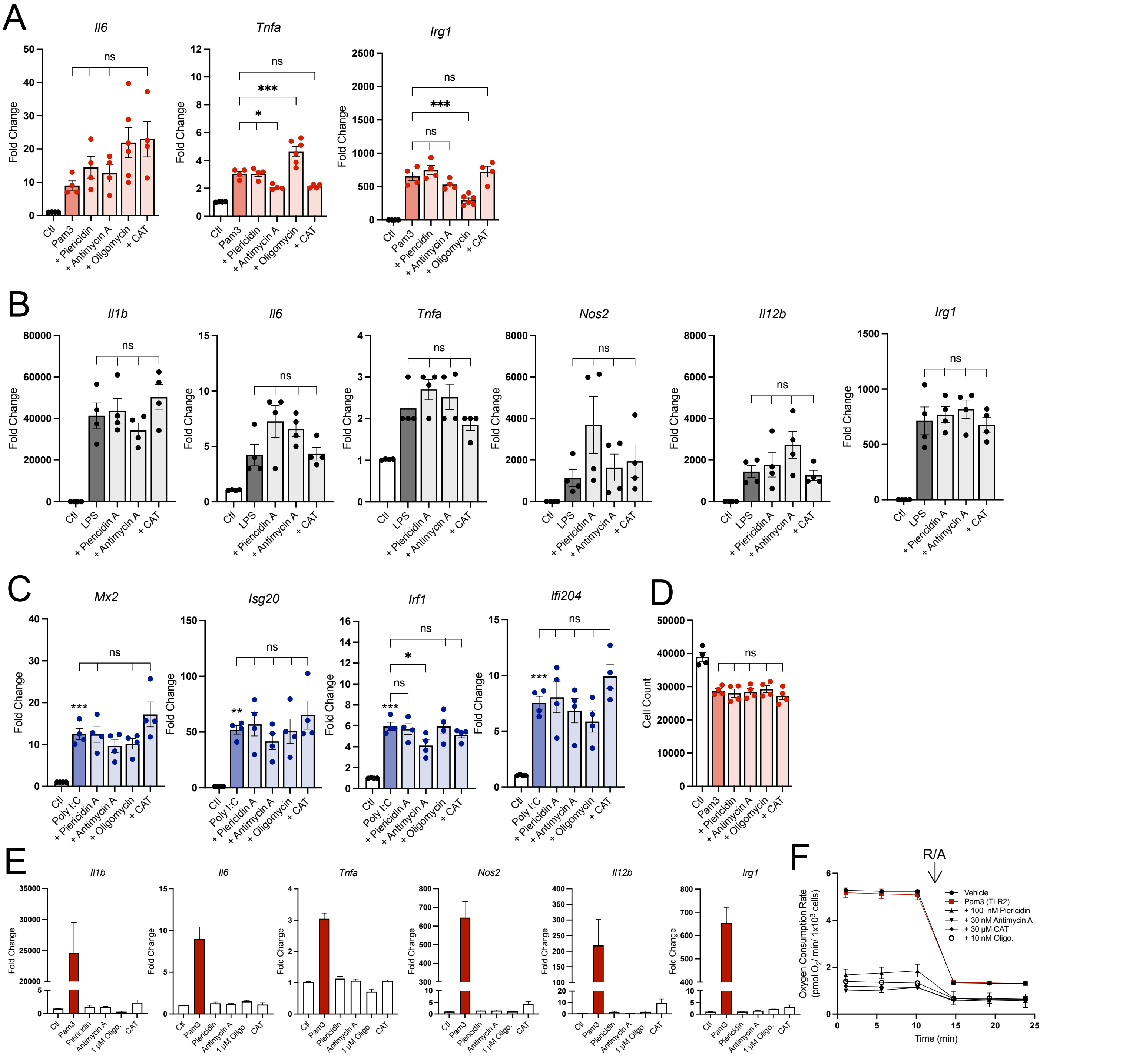

Appendix Figure S8: Validation of mitochondrial effector compounds across multiple MyD88- and TRIF-linked stimuli. A-C) Pro-inflammatory gene expression of control (Ctl) and BMDMs treated with Pam3 (n = 4-6) (**A**), LPS (n = 4) (**B**), or Poly I:C with mitochondrial effector compounds for 24 hr. (n = 4) (**C**). **D**) Cell counts via high-content imaging of Hoechst stained control (Ctl) and BMDMs treated with Pam3 with mitochondrial effector compounds for 24 hr. (n = 4). **E**) Pro-inflammatory gene expression of BMDMs treated with control (Ctl), Pam3 alone, or mitochondrial effector compounds alone for 24 hr. CAT, carboxyatractyloside (n = 3-4). **F**) Oxygen consumption rates for control (Ctl) and BMDMs treated with Pam3 with mitochondrial effector compounds for 24 hr. R/A, rotenone/antimycin A (n = 1 biological with 5 technical replicates). All data are mean ± SEM with statistical analysis conducted on data from biological replicates, each of which included multiple technical replicates, unless otherwise indicated. Statistical analysis for (**A-D**) was performed as an ordinary one-way, ANOVA followed by Tukey's *post hoc* multiple comparisons test.

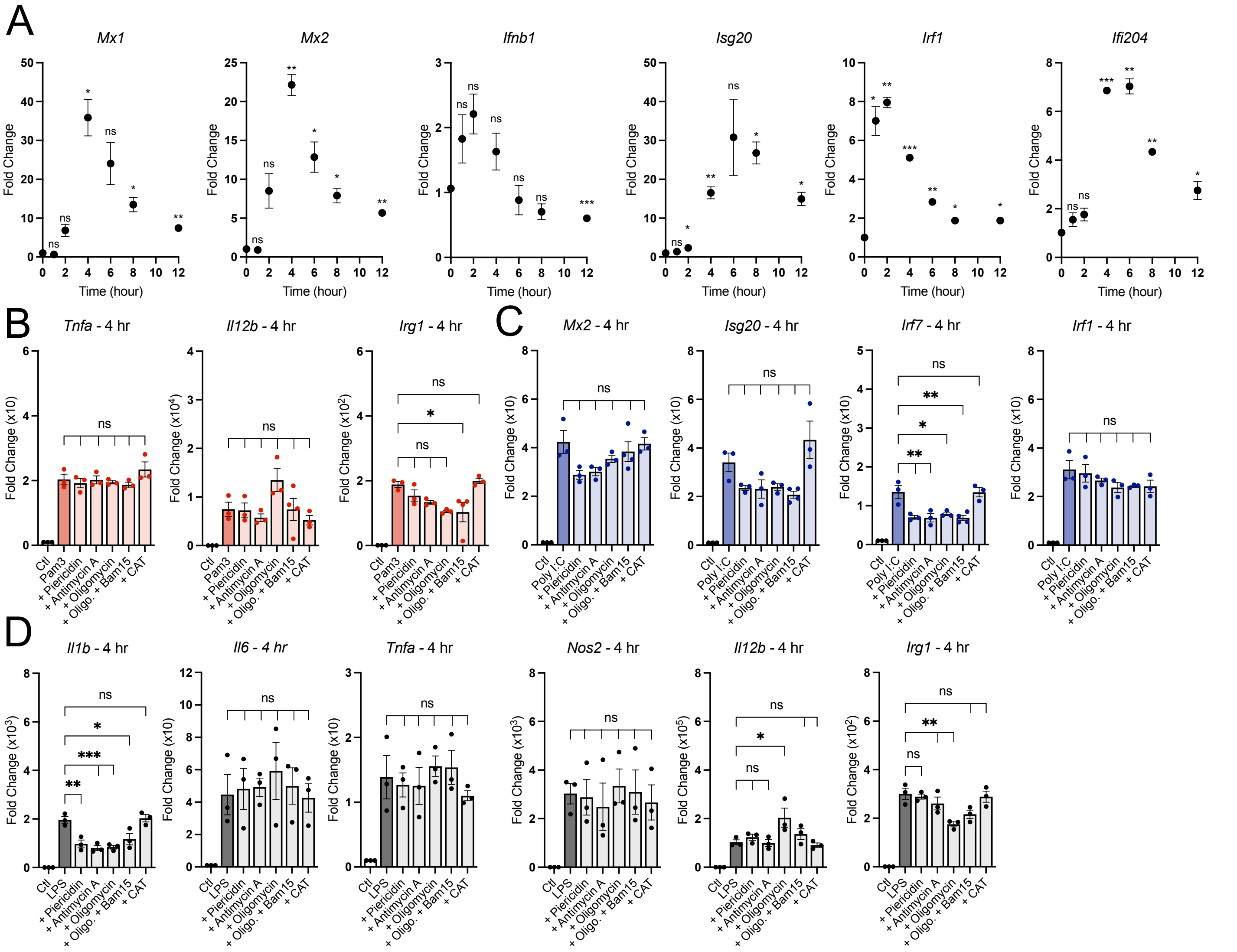

Appendix Figure S9: At 4 hr., there is no link between mitochondrial inhibition and the induction of pro-inflammatory gene expression across stimuli. A) Pro-inflammatory gene expression in BMDMs treated with 50 ng/mL LPS across multiple timepoints relative to control (Ctl) (n = 3). **B**-**D**) Pro-inflammatory gene expression of control (Ctl) and BMDMs treated with Pam3 (n = 4-6) (**B**), Poly I:C (**C**), or 10 ng/mL LPS with mitochondrial effector compounds for 4 hr. (n = 3) (**D**). All data are mean ± SEM with statistical analysis conducted on data from biological replicates, each of which included multiple technical replicates, unless otherwise indicated. Statistical analysis for (**A**) was performed as a paired, two-tailed t-test. Statistical analysis for (**B-D**) was performed as an ordinary one-way, ANOVA followed by Tukey's *post hoc* multiple comparisons test.

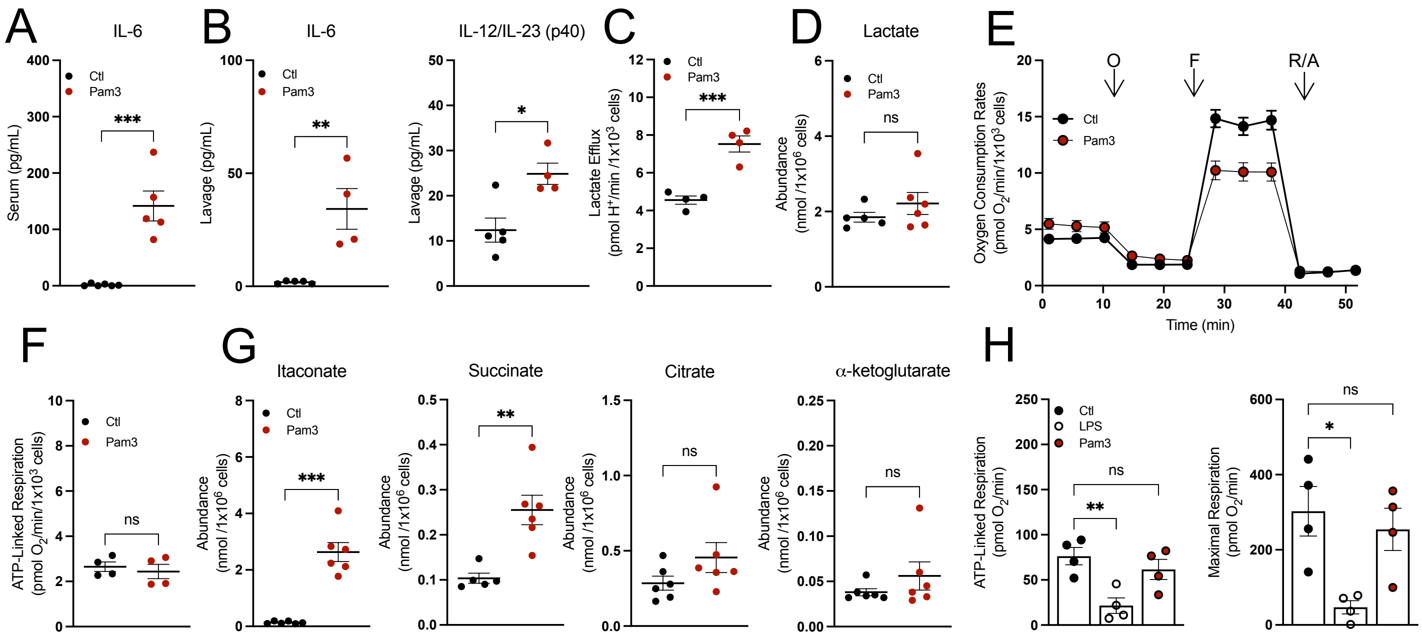

Appendix Figure S10: Peritoneal macrophages activated *in vivo* with Pam3 do not require respiratory inhibition. A&B) Cytokine levels from serum (n = 5-6) (**A**) and lavage fluid from mice intraperitoneally injected with PBS or Pam3 for 24 hr. (n = 4-6) (**B**). **C**) Lactate efflux rates for peritoneal macrophages isolated from mice intraperitoneally injected with PBS or Pam3 for 24 hr. (n = 4). **D**) Intracellular lactate abundance from peritoneal macrophages isolated from mice intraperitoneally injected with PBS or Pam3 for 24 hr. (n = 5-6). **E**) Representative oxygen consumption trace with peritoneal macrophages isolated from mice intraperitoneally injected with PBS or Pam3 for 24 hr. Where not visible, error bars are obscured by the symbol. O, oligomycin; F, carbonyl cyanide-p-trifluoromethoxyphenylhydrazone (FCCP); R/A, rotenone/antimycin A (n = 1 biological replicate with 5 technical replicates). **F**) ATP-Linked respiration for peritoneal macrophages isolated from mice intraperitoneally injected with PBS or Pam3 for 24 hr. (n = 4). **G**) Intracellular itaconate, succinate, citrate and α-ketoglutarate abundances from peritoneal macrophages isolated from mice intraperitoneally injected with PBS or Pam3 for 24 hr. (n = 6). **H**) ATP-linked and maximal respiration rates for peritoneal macrophages treated *in vitro* with 50 ng/mL LPS or 50 ng/mL Pam3 for 24 hr. (n = 4). All data are mean ± SEM with statistical analysis conducted on data from biological replicates, each of which included multiple technical replicates, unless otherwise indicated. Statistical analysis for (**A-D**) and (**F-G**) was performed as an an unpaired, two-tailed t-test. Statistical analysis for (**H**) was performed as an ordinary one-way, ANOVA followed by Tukey's *post hoc* multiple comparisons test.

Appendix Table S1

13C Gluc - alpha-ketoglutarate MID						
	**M0**	**M1**	**M2**	**M3**	**M4**	**M5**
**NT M1**	75.67	7.62	14.14	1.95	1.30	-0.61
**NT M2**	79.28	6.68	14.91	-0.54	-0.08	-0.28
**NT M3**	74.90	3.96	16.51	3.04	2.12	-0.23
**NT M4**	77.90	4.99	14.43	2.22	1.03	-0.49
	**M0**	**M1**	**M2**	**M3**	**M4**	**M5**
**Pam3 M1**	64.73	3.44	25.52	3.21	2.77	0.47
**Pam3 M2**	62.20	2.90	27.56	3.34	3.50	0.53
**Pam3 M3**	74.19	1.27	21.50	1.46	1.58	0.13
**Pam3 M4**	70.77	1.83	24.32	2.09	1.09	-0.12
	**M0**	**M1**	**M2**	**M3**	**M4**	**M5**
**PIC M1**	72.05	3.86	18.51	2.90	2.63	0.50
**PIC M2**	69.72	4.82	19.52	4.10	1.99	0.18
**PIC M3**	74.05	6.38	16.17	2.97	1.20	-0.70
**PIC M4**	73.93	5.19	16.62	2.26	2.21	0.08
	**M0**	**M1**	**M2**	**M3**	**M4**	**M5**
**Both M1**	93.87	1.36	4.16	0.83	-0.03	-0.22
**Both M2**	99.67	-0.92	1.93	-0.49	-0.25	0.04
**Both M3**	101.16	-0.25	0.31	-1.43	0.15	0.07
**Both M4**	97.10	0.80	2.40	0.22	-0.68	0.14

13C Gluc - Aspartate MID					
	**M0**	**M1**	**M2**	**M3**	**M4**
**NT M1**	77.70	8.03	11.35	2.78	0.18
**NT M2**	79.51	8.16	9.84	2.51	-0.01
**NT M3**	75.33	7.68	12.91	3.72	0.37
**NT M4**	78.01	7.73	10.99	3.10	0.27
	**M0**	**M1**	**M2**	**M3**	**M4**
**Pam3 M1**	76.03	4.19	14.80	4.44	0.58
**Pam3 M2**	70.70	5.86	16.61	6.10	0.77
**Pam3 M3**	83.70	2.58	10.28	3.20	0.27
**Pam3 M4**	81.14	3.30	11.58	3.45	0.52
	**M0**	**M1**	**M2**	**M3**	**M4**
**PIC M1**	77.44	6.90	12.26	3.14	0.32
**PIC M2**	77.63	6.74	12.18	3.30	0.20
**PIC M3**	80.59	6.28	10.73	2.25	0.11
**PIC M4**	79.72	6.53	11.04	2.67	0.06
	**M0**	**M1**	**M2**	**M3**	**M4**
**Both M1**	93.13	-0.36	2.69	4.59	0.00
**Both M2**	93.30	0.01	0.91	5.79	0.04

**Both M3**	94.85	-0.76	1.09	5.25	-0.25
**Both M4**	92.29	0.07	2.69	5.11	-0.05

13C Gluc - Citrate 459 MID

	**M0**	**M1**	**M2**	**M3**	**M4**	**M5**	**M6**
**NT M1**	60.95	6.67	25.20	3.67	2.90	0.82	-0.13
**NT M2**	66.08	5.78	23.14	2.52	2.30	0.77	-0.52
**NT M3**	56.25	5.33	28.62	4.67	4.40	0.90	-0.17
**NT M4**	59.76	7.34	25.57	3.92	2.77	0.75	0.02
	**M0**	**M1**	**M2**	**M3**	**M4**	**M5**	**M6**
**Pam3 M1**	41.72	2.87	41.69	3.92	7.87	2.01	0.31
**Pam3 M2**	39.95	4.12	39.66	4.85	8.72	2.62	0.27
**Pam3 M3**	49.51	2.21	40.15	1.90	4.92	1.22	0.12
**Pam3 M4**	47.49	2.88	40.94	2.69	4.73	1.64	-0.28
	**M0**	**M1**	**M2**	**M3**	**M4**	**M5**	**M6**
**PIC M1**	57.88	5.11	27.61	4.32	3.82	1.16	0.06
**PIC M2**	56.81	5.76	28.39	4.11	3.71	0.95	0.30
**PIC M3**	61.63	5.59	26.16	3.43	2.49	0.59	0.29
**PIC M4**	61.15	6.32	25.92	2.94	3.00	0.63	0.08
	**M0**	**M1**	**M2**	**M3**	**M4**	**M5**	**M6**
**Both M1**	69.10	0.13	23.85	4.30	1.25	1.56	-0.06
**Both M2**	80.39	-0.95	10.92	8.49	0.23	1.06	-0.15
**Both M3**	81.85	-1.68	11.54	7.92	-0.46	0.88	-0.04
**Both M4**	74.67	0.06	16.77	6.85	0.22	1.39	0.04

13C Gluc - Fumarate MID

	**M0**	**M1**	**M2**	**M3**	**M4**
**NT M1**	84.68	6.18	7.06	2.27	-0.16
**NT M2**	86.35	4.72	7.60	2.20	-0.58
**NT M3**	80.15	4.44	12.12	3.56	-0.12
**NT M4**	80.70	6.72	10.29	2.50	0.10
	**M0**	**M1**	**M2**	**M3**	**M4**
**Pam3 M1**	84.91	0.05	12.58	2.17	0.42
**Pam3 M2**	76.69	1.83	15.29	5.64	0.77
**Pam3 M3**	92.27	-0.36	6.34	1.56	0.24
**Pam3 M4**	88.66	0.24	9.04	1.83	0.25
	**M0**	**M1**	**M2**	**M3**	**M4**
**PIC M1**	86.28	1.18	9.57	3.56	-0.31
**PIC M2**	77.38	6.65	11.63	4.20	0.45
**PIC M3**	82.19	4.52	10.81	2.24	0.97

	M0	M1	M2	M3	M4
**PIC M4**	80.51	6.02	10.67	2.70	0.63

	M0	M1	M2	M3	M4
**Both M1**	97.09	-2.23	1.77	3.49	-0.03
**Both M2**	93.50	0.88	1.40	6.53	-0.17
**Both M3**	96.10	-1.78	1.47	4.97	-0.25
**Both M4**	94.55	-1.52	1.92	5.32	-0.04

13C Gluc - Itaconate MID

	M0	M1	M2	M3	M4	M5
**NT M1**	65.51	21.52	8.77	4.51	0.71	-0.90
**NT M2**	71.15	18.82	8.34	3.00	-0.98	-0.15
**NT M3**	59.87	24.64	9.85	5.55	0.55	-0.79
**NT M4**	67.60	21.21	8.34	3.52	0.01	-0.46

	M0	M1	M2	M3	M4	M5
**Pam3 M1**	63.30	26.14	4.80	5.39	0.88	-0.34
**Pam3 M2**	68.94	21.16	4.73	4.97	0.67	-0.31
**Pam3 M3**	62.49	27.55	4.65	5.17	0.74	-0.43
**Pam3 M4**	61.47	28.04	4.89	5.35	0.82	-0.40

	M0	M1	M2	M3	M4	M5
**PIC M1**	63.69	22.81	8.79	4.91	0.37	-0.39
**PIC M2**	65.47	22.01	8.24	4.61	0.24	-0.39
**PIC M3**	68.10	20.76	7.79	3.90	0.00	-0.38
**PIC M4**	67.57	20.97	8.03	4.02	-0.03	-0.38

	M0	M1	M2	M3	M4	M5
**Both M1**	78.43	14.11	1.97	5.84	0.08	-0.35
**Both M2**	86.29	6.23	1.68	6.70	-0.43	-0.35
**Both M3**	86.93	6.33	0.82	6.82	-0.42	-0.34
**Both M4**	82.71	9.74	1.84	6.25	-0.05	-0.41

13C Gluc - Malate MID

	M0	M1	M2	M3	M4
**NT M1**	79.23	6.86	10.84	2.85	0.30
**NT M2**	83.43	6.60	7.51	2.79	0.03
**NT M3**	75.30	6.94	13.36	4.10	0.11
**NT M4**	79.94	6.32	10.85	3.04	-0.13

	M0	M1	M2	M3	M4
**Pam3 M1**	79.73	2.89	13.62	3.41	0.36
**Pam3 M2**	73.33	4.70	16.18	5.13	0.73
**Pam3 M3**	90.14	1.12	6.99	1.47	0.37
**Pam3 M4**	86.14	2.20	9.13	2.50	0.01

	M0	M1	M2	M3	M4
**PIC M1**	75.84	7.16	12.73	4.03	0.30
**PIC M2**	75.50	7.66	13.47	3.40	0.40
**PIC M3**	80.45	5.94	10.27	3.05	0.27
**PIC M4**	78.56	6.59	10.94	3.52	0.38
	**M0**	**M1**	**M2**	**M3**	**M4**
**Both M1**	92.93	-0.32	3.15	4.18	0.12
**Both M2**	93.96	-0.01	0.63	6.22	-0.71
**Both M3**	96.27	-1.75	0.38	5.17	-0.18
**Both M4**	92.61	-0.13	1.71	5.90	-0.19

13C Gluc - Succinate MID

	M0	M1	M2	M3	M4
**NT M1**	86.77	6.28	6.11	0.70	0.28
**NT M2**	84.99	8.19	6.74	0.35	-0.08
**NT M3**	84.44	4.88	7.85	3.18	-0.12
**NT M4**	83.61	6.00	9.09	1.36	0.05
	**M0**	**M1**	**M2**	**M3**	**M4**
**Pam3 M1**	75.21	1.71	18.01	4.06	1.11
**Pam3 M2**	66.48	3.65	24.23	4.28	1.38
**Pam3 M3**	86.85	1.24	10.02	1.66	0.27
**Pam3 M4**	82.11	1.94	13.66	1.96	0.39
	**M0**	**M1**	**M2**	**M3**	**M4**
**PIC M1**	79.26	6.15	10.39	3.76	0.49
**PIC M2**	75.49	6.97	13.94	3.19	0.40
**PIC M3**	78.70	6.65	12.10	2.31	0.29
**PIC M4**	78.17	6.37	12.51	2.65	0.29
	**M0**	**M1**	**M2**	**M3**	**M4**
**Both M1**	93.73	-0.09	5.63	0.43	0.34
**Both M2**	97.40	-0.28	2.47	0.39	0.14
**Both M3**	98.93	-0.20	0.94	0.37	0.06
**Both M4**	97.26	-0.90	2.98	0.49	0.24

13C Q - alpha-ketoglutarate MID

	M0	M1	M2	M3	M4	M5
**NT M1**	37.40	11.82	5.39	16.33	-0.31	30.87
**NT M2**	39.20	10.95	5.91	15.59	0.78	28.54
**NT M3**	32.59	9.83	6.28	17.27	0.43	35.71
**NT M4**	33.25	9.69	7.68	16.32	1.24	33.91
	**M0**	**M1**	**M2**	**M3**	**M4**	**M5**
**Pam3 M1**	52.16	3.51	3.78	11.45	0.75	30.54
**Pam3 M2**	56.52	4.26	3.61	11.00	0.60	26.01
**Pam3 M3**	60.17	1.70	1.62	8.60	0.73	28.77
**Pam3 M4**	59.08	2.19	2.75	9.36	0.95	27.61
	**M0**	**M1**	**M2**	**M3**	**M4**	**M5**
**PIC M1**	28.58	10.12	5.88	18.20	1.31	37.74
**PIC M2**	30.51	10.67	4.49	19.35	0.30	37.53
**PIC M3**	29.39	12.83	6.08	16.88	1.05	36.70
**PIC M4**	30.75	11.42	2.18	18.31	0.02	38.49
	**M0**	**M1**	**M2**	**M3**	**M4**	**M5**
**Both M1**	40.08	1.47	-0.46	6.52	1.02	55.28
**Both M2**	43.87	0.67	-0.22	2.97	1.33	55.36
**Both M3**	48.23	-1.64	-0.64	2.25	0.84	54.44
**Both M4**	44.88	-0.18	0.38	3.08	1.54	57.36

13C Q - Aspartate MID

	M0	M1	M2	M3	M4
**NT M1**	43.40	13.90	15.79	3.01	25.12
**NT M2**	47.02	13.30	14.59	2.76	23.59
**NT M3**	42.61	12.30	15.96	2.84	27.76
**NT M4**	40.21	13.62	16.10	3.35	28.30
	**M0**	**M1**	**M2**	**M3**	**M4**
**Pam3 M1**	54.24	5.08	10.66	4.08	27.29
**Pam3 M2**	46.17	7.87	13.53	4.60	29.46
**Pam3 M3**	61.69	3.58	8.80	3.32	23.52
**Pam3 M4**	54.10	5.65	10.42	4.41	26.79
	**M0**	**M1**	**M2**	**M3**	**M4**
**PIC M1**	31.22	13.91	18.55	3.09	34.66
**PIC M2**	33.77	13.68	18.16	3.11	33.16
**PIC M3**	33.62	13.99	17.89	2.69	33.53
**PIC M4**	34.38	13.39	17.83	2.51	33.62
	**M0**	**M1**	**M2**	**M3**	**M4**
**Both M1**	61.72	0.01	4.17	2.77	32.98
**Both M2**	70.89	0.50	1.86	2.29	25.59

**Both M3**	68.80	-0.03	2.29	2.42	28.05
**Both M4**	67.96	-0.31	3.48	2.31	28.35

13C Q - Citrate 459 MID

	M0	M1	M2	M3	M4	M5	M6
**NT M1**	48.49	12.91	13.07	4.09	19.45	1.82	0.16
**NT M2**	51.32	12.51	12.26	4.04	18.67	1.70	-0.10
**NT M3**	44.90	12.09	13.74	4.51	22.67	1.70	0.12
**NT M4**	42.98	13.21	14.14	4.46	23.54	1.72	-0.06
	**M0**	**M1**	**M2**	**M3**	**M4**	**M5**	**M6**
**Pam3 M1**	56.79	4.81	9.50	5.30	19.89	3.69	0.08
**Pam3 M2**	54.81	6.80	9.67	5.68	19.17	4.02	-0.12
**Pam3 M3**	64.02	3.06	7.51	4.02	17.34	4.28	-0.32
**Pam3 M4**	59.21	4.18	8.63	4.74	19.92	3.52	-0.20
	**M0**	**M1**	**M2**	**M3**	**M4**	**M5**	**M6**
**PIC M1**	33.16	13.95	15.95	5.75	27.27	3.90	0.05
**PIC M2**	33.64	13.81	15.87	5.82	27.13	3.98	-0.34
**PIC M3**	35.02	13.75	15.84	5.16	27.29	2.91	-0.12
**PIC M4**	33.15	13.84	16.30	5.54	27.73	3.96	-0.38
	**M0**	**M1**	**M2**	**M3**	**M4**	**M5**	**M6**
**Both M1**	54.11	0.42	4.15	3.46	38.94	-1.04	-0.20
**Both M2**	64.18	-0.39	2.58	2.87	31.67	-0.77	-0.28
**Both M3**	62.20	-0.81	2.63	3.18	34.16	-1.12	-0.43
**Both M4**	61.85	0.36	2.49	2.88	33.22	-0.61	-0.18

13C Q - Fumarate MID

	M0	M1	M2	M3	M4
**NT M1**	62.72	7.27	11.45	2.33	16.45
**NT M2**	57.23	6.17	13.44	3.46	20.30
**NT M3**	46.38	10.83	13.96	3.24	27.13
**NT M4**	45.49	12.26	13.87	3.32	27.30
	**M0**	**M1**	**M2**	**M3**	**M4**
**Pam3 M1**	70.97	3.00	5.36	2.57	19.25
**Pam3 M2**	60.74	4.95	9.69	3.65	21.99
**Pam3 M3**	81.99	0.03	3.53	2.18	13.23
**Pam3 M4**	74.40	1.09	6.09	2.75	16.76
	**M0**	**M1**	**M2**	**M3**	**M4**
**PIC M1**	49.29	9.86	10.01	3.20	29.82
**PIC M2**	37.82	15.48	15.05	3.54	30.19
**PIC M3**	40.23	12.83	15.81	2.15	30.96

**PIC M4**	40.20	12.13	16.88	2.46	31.12

	**M0**	**M1**	**M2**	**M3**	**M4**
**Both M1**	73.73	-0.44	1.75	2.41	23.06
**Both M2**	68.91	0.60	2.80	2.36	26.72
**Both M3**	72.50	-0.94	0.83	2.38	27.19
**Both M4**	69.48	0.22	1.09	2.70	28.68

13C Q - Itaconate MID

	**M0**	**M1**	**M2**	**M3**	**M4**	**M5**
**NT M1**	41.56	13.74	14.27	6.21	25.74	-1.23
**NT M2**	47.06	12.90	13.18	5.52	23.20	-0.77
**NT M3**	42.31	11.35	14.36	6.32	27.88	-1.63
**NT M4**	41.34	13.25	14.26	5.56	27.27	-1.07

	**M0**	**M1**	**M2**	**M3**	**M4**	**M5**
**Pam3 M1**	59.67	3.91	6.97	5.61	25.33	-1.32
**Pam3 M2**	66.61	3.82	6.15	4.91	19.86	-1.18
**Pam3 M3**	59.00	3.47	6.94	5.74	26.58	-1.55
**Pam3 M4**	57.00	4.16	7.54	5.79	27.24	-1.52

	**M0**	**M1**	**M2**	**M3**	**M4**	**M5**
**PIC M1**	33.38	13.31	15.40	7.16	32.37	-1.33
**PIC M2**	36.93	12.10	14.20	7.33	31.18	-1.45
**PIC M3**	37.06	12.89	14.77	6.49	30.51	-1.36
**PIC M4**	36.82	12.66	14.76	6.68	30.84	-1.43

	**M0**	**M1**	**M2**	**M3**	**M4**	**M5**
**Both M1**	58.02	0.61	3.95	4.06	35.65	-2.13
**Both M2**	66.85	0.28	3.02	3.56	28.32	-1.90
**Both M3**	66.43	-0.30	2.24	3.15	30.61	-2.04
**Both M4**	64.41	0.20	2.58	3.75	31.26	-2.06

13C Q - Malate MID

	**M0**	**M1**	**M2**	**M3**	**M4**
**NT M1**	45.70	12.44	14.89	3.02	25.63
**NT M2**	50.43	10.69	13.96	2.92	23.12
**NT M3**	42.70	11.92	15.01	3.04	28.76
**NT M4**	39.77	13.55	16.00	3.38	29.03

	**M0**	**M1**	**M2**	**M3**	**M4**
**Pam3 M1**	66.64	3.25	7.15	3.30	20.86
**Pam3 M2**	58.54	5.37	9.95	3.50	24.04
**Pam3 M3**	78.16	1.37	4.30	2.26	14.79
**Pam3 M4**	69.68	3.18	6.63	2.65	19.19

	M0	M1	M2	M3	M4
**PIC M1**	31.44	13.96	18.65	2.57	35.05
**PIC M2**	33.91	13.46	17.84	2.86	33.60
**PIC M3**	33.94	14.51	17.30	2.27	33.36
**PIC M4**	33.17	13.80	17.79	2.10	35.26

	M0	M1	M2	M3	M4
**Both M1**	59.97	0.71	3.39	2.62	35.02
**Both M2**	67.22	0.40	1.56	2.53	30.11
**Both M3**	68.47	0.11	1.94	2.17	29.04
**Both M4**	64.14	0.51	1.84	2.80	32.47

13C Q - Succinate MID

	M0	M1	M2	M3	M4
**NT M1**	71.15	4.09	9.34	0.85	16.16
**NT M2**	69.65	4.49	9.24	0.72	17.20
**NT M3**	59.58	8.30	10.23	0.82	22.20
**NT M4**	49.37	9.85	13.34	0.99	28.23

	M0	M1	M2	M3	M4
**Pam3 M1**	51.78	8.15	13.63	-0.58	28.30
**Pam3 M2**	55.42	4.00	10.89	1.67	29.88
**Pam3 M3**	69.03	2.58	6.40	0.83	22.31
**Pam3 M4**	57.09	4.08	10.16	0.90	29.50

	M0	M1	M2	M3	M4
**PIC M1**	35.42	13.38	17.75	1.22	33.86
**PIC M2**	35.85	13.73	18.06	1.27	33.14
**PIC M3**	33.80	13.79	17.97	1.06	35.13
**PIC M4**	30.23	14.00	19.01	1.41	37.70

	M0	M1	M2	M3	M4
**Both M1**	53.47	0.31	5.26	1.48	44.31
**Both M2**	62.69	-0.37	3.04	1.26	35.48
**Both M3**	58.13	-0.31	3.03	1.36	40.38
**Both M4**	51.07	0.16	3.94	0.63	46.81

Abundance (nmol/1e6 cells)

AKG

Replicate	NT	Pam3	PIC	Pam3 + PIC
Mouse 1	0.083	0.765	0.162	0.094
Mouse 2	0.089	1.087	0.163	0.087
Mouse 3	0.137	0.803	0.136	0.071
Mouse 4	0.144	1.06	0.165	0.14

Aspartate

Replicate	NT	Pam3	PIC	Pam3 + PIC
Mouse 1	4.901	3.462	2.263	0.953
Mouse 2	4.292	2.535	2.568	0.831
Mouse 3	3.688	3.609	2.216	0.833
Mouse 4	4.446	2.839	2.422	0.848

Citrate

Replicate	NT	Pam3	PIC	Pam3 + PIC
Mouse 1	0.44	0.878	0.563	1.052
Mouse 2	0.385	0.861	0.746	0.89
Mouse 3	0.42	1.027	0.55	1.194
Mouse 4	0.573	0.977	0.658	1.049

Fumarate

Replicate	NT	Pam3	PIC	Pam3 + PIC
Mouse 1	0.130	0.616	0.175	0.177
Mouse 2	0.096	0.31	0.129	0.137
Mouse 3	0.136	0.826	0.13	0.129
Mouse 4	0.137	0.648	0.134	0.152

Itaconate

Replicate	NT	Pam3	PIC	Pam3 + PIC
Mouse 1	0.557	20.485	12.266	9.997
Mouse 2	0.442	20.588	15.02	7.029
Mouse 3	0.297	11.259	13.041	7.752
Mouse 4	0.542	16.857	14.883	9.704

Malate

Replicate	NT	Pam3	PIC	Pam3 + PIC
Mouse 1	0.518	2.508	0.708	0.655
Mouse 2	0.445	1.619	0.774	0.714
Mouse 3	0.653	3.656	0.653	0.59

**Mouse 4**	0.723	3.032	0.752	0.806

**Succinate**				
**Replicate**	**NT**	**Pam3**	**PIC**	**Pam3 + PIC**
**Mouse 1**	0.293	3.594	1.918	1.945
**Mouse 2**	0.194	2.884	1.835	0.879
**Mouse 3**	0.183	2.784	1.747	1.078
**Mouse 4**	0.278	4.255	2.453	1.655
