# Supplementary material for: Pro-inflammatory macrophage activation does not require inhibition of oxidative phosphorylation
Source: EMBO Rep. 2025 Jan 3;26(4):982–1002. doi: 10.1038/s44319-024-00351-y (PMC11850891; doi:10.1038/s44319-024-00351-y)
Supplement: Supplementary file 3 — Source data Fig. 1 [file 44319_2024_351_MOESM3_ESM.zip › README FIG 1.rtf]

Figure 1 includes data from several orthogonal measurements of mitochondrial bioenergetics in BMDMs treated with multiple different pro-inflammatory stimuli and combinations of stimuli. 
