# Supplementary figures and images for: Pro-inflammatory macrophage activation does not require inhibition of oxidative phosphorylation
Source: EMBO Rep. 2025 Jan 3;26(4):982–1002. doi: 10.1038/s44319-024-00351-y (PMC11850891; doi:10.1038/s44319-024-00351-y)

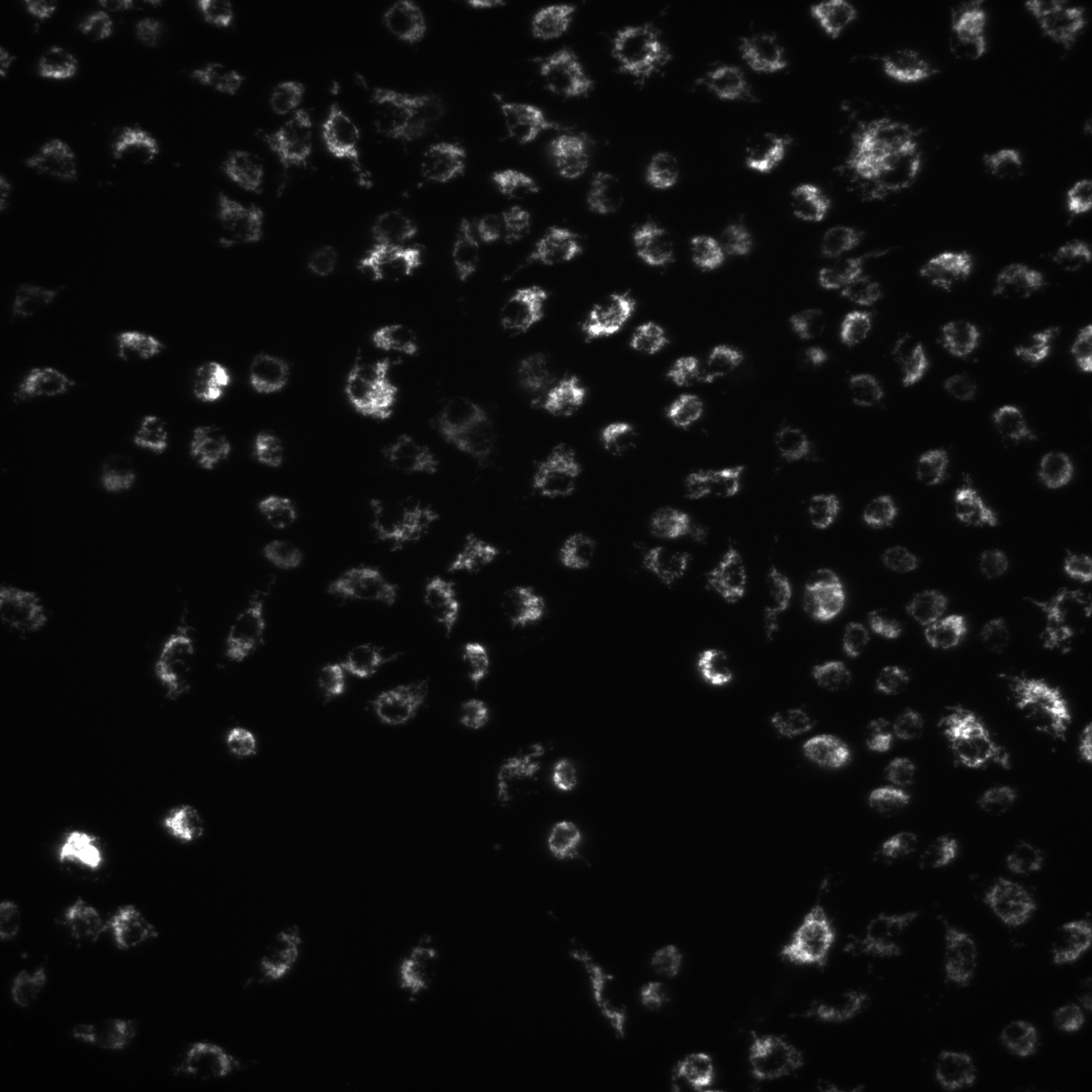

Supplement: Supplementary file 3 — Source data Fig. 1 [file 44319_2024_351_MOESM3_ESM.zip › 1H/B3.tif]

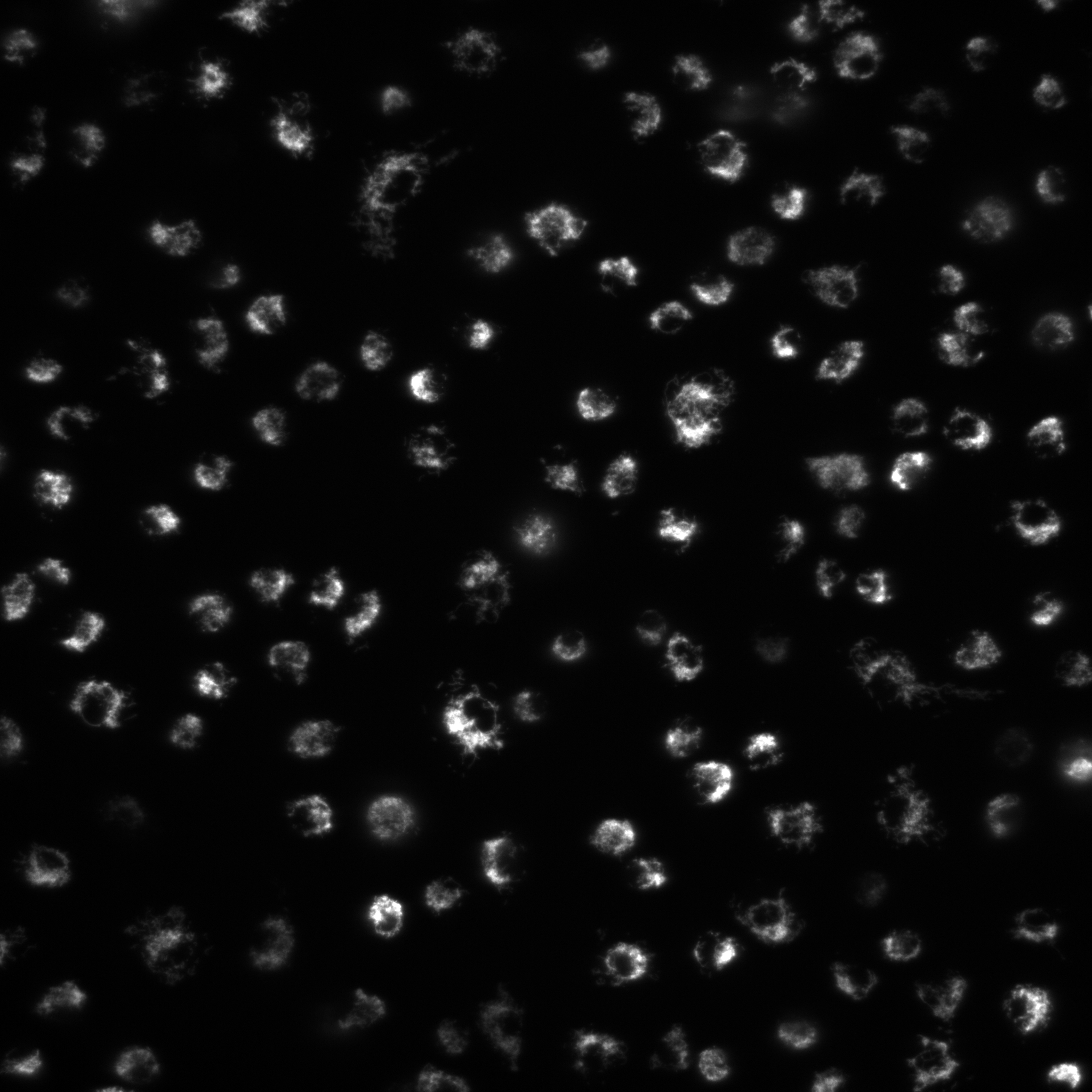

Supplement: Supplementary file 3 — Source data Fig. 1 [file 44319_2024_351_MOESM3_ESM.zip › 1H/C3.tif]

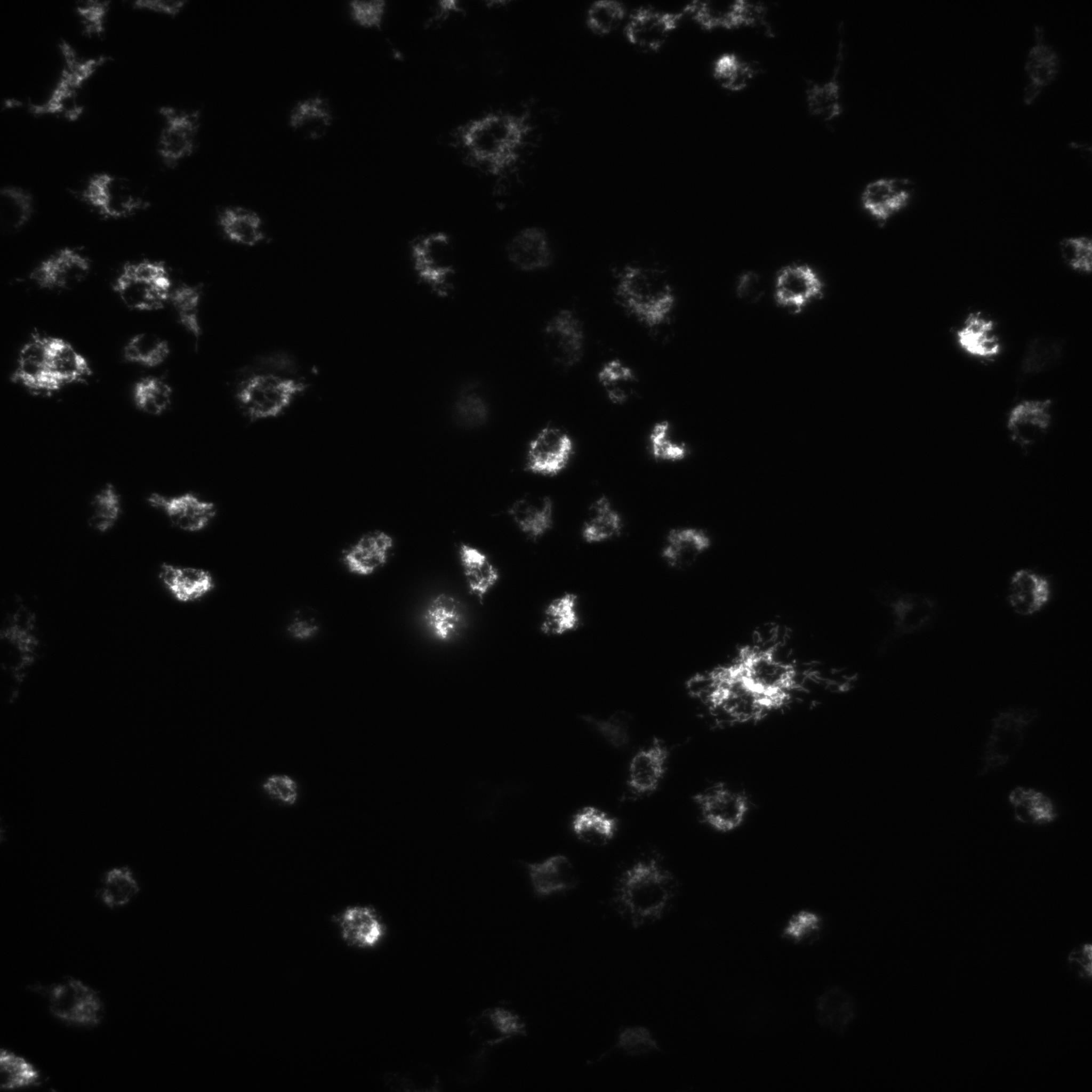

Supplement: Supplementary file 3 — Source data Fig. 1 [file 44319_2024_351_MOESM3_ESM.zip › 1H/D3.tif]

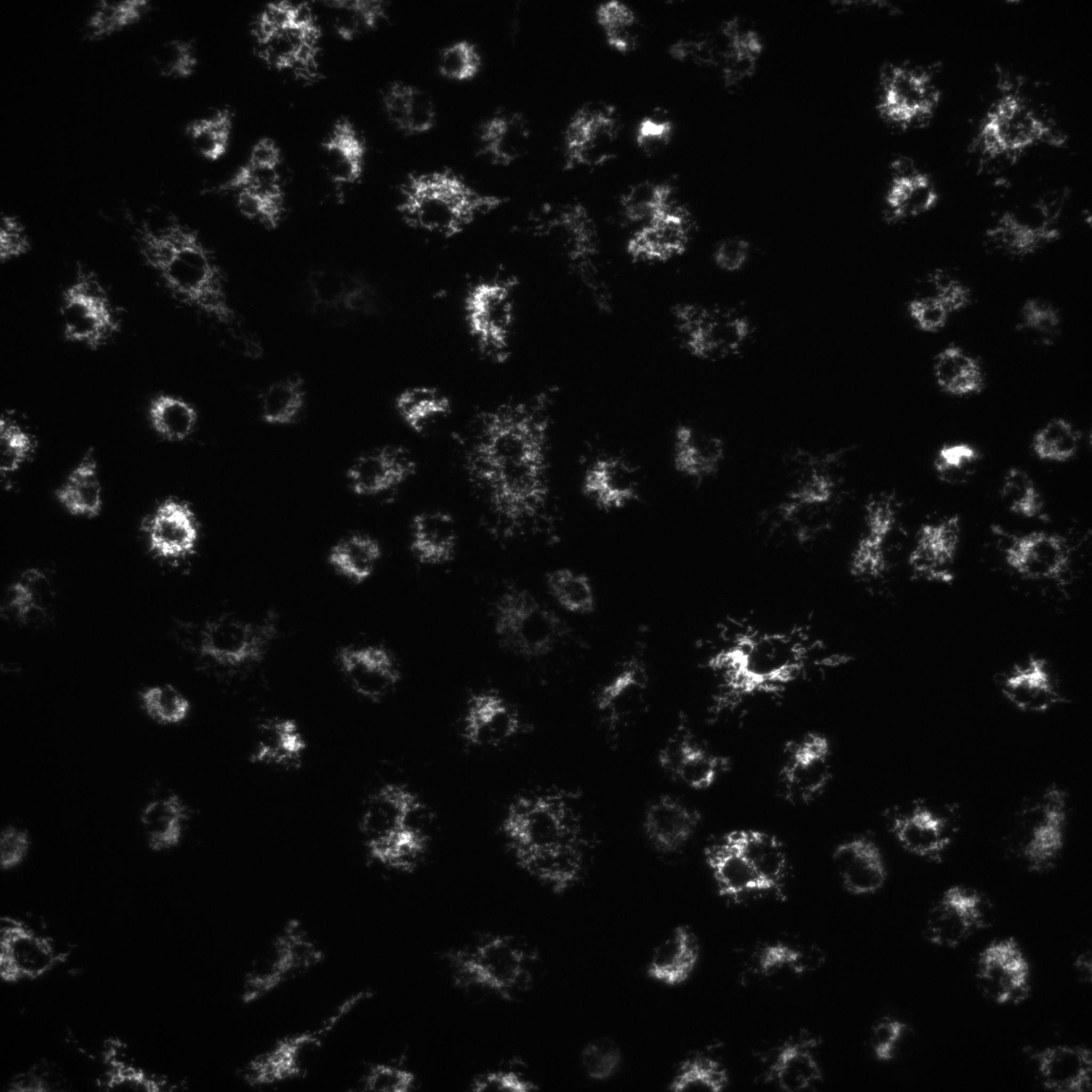

Supplement: Supplementary file 3 — Source data Fig. 1 [file 44319_2024_351_MOESM3_ESM.zip › 1H/E4.tif]

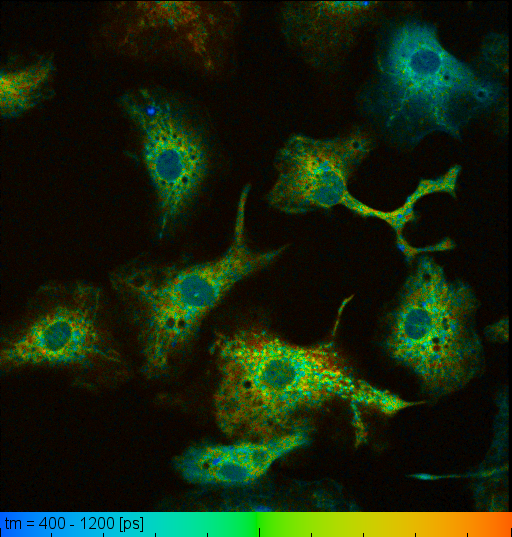

Supplement: Supplementary file 3 — Source data Fig. 1 [file 44319_2024_351_MOESM3_ESM.zip › 1J/Both_08_0_color_image.tif]

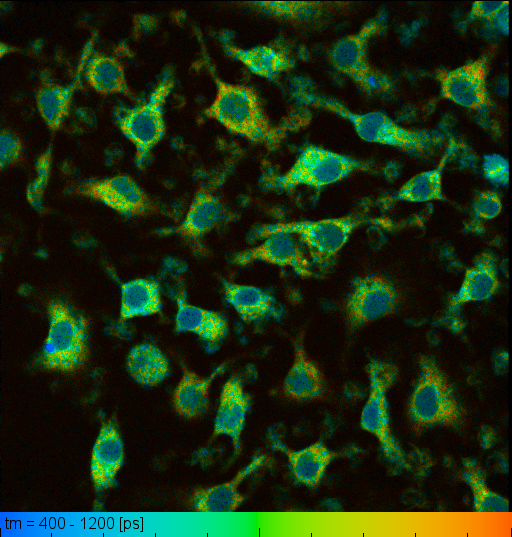

Supplement: Supplementary file 3 — Source data Fig. 1 [file 44319_2024_351_MOESM3_ESM.zip › 1J/control_03_0_color_image.tif]

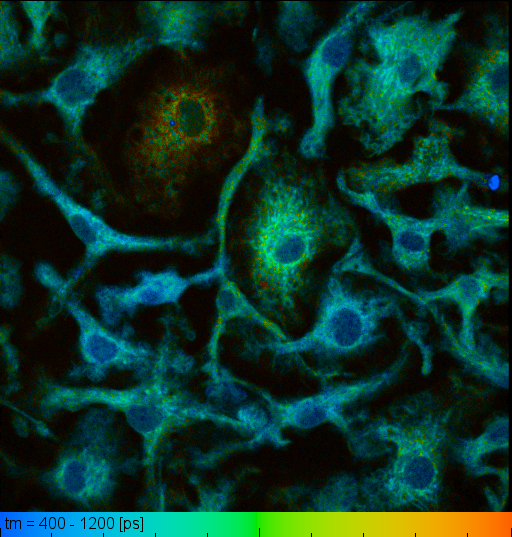

Supplement: Supplementary file 3 — Source data Fig. 1 [file 44319_2024_351_MOESM3_ESM.zip › 1J/PAM3_02_0_color_image.tif]

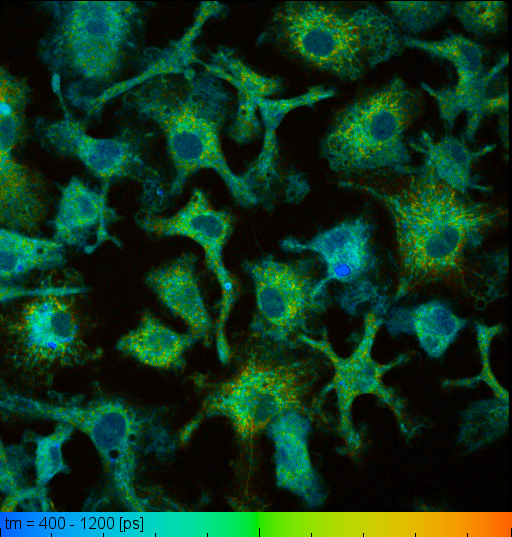

Supplement: Supplementary file 3 — Source data Fig. 1 [file 44319_2024_351_MOESM3_ESM.zip › 1J/PIC_02_0_color_image.tif]

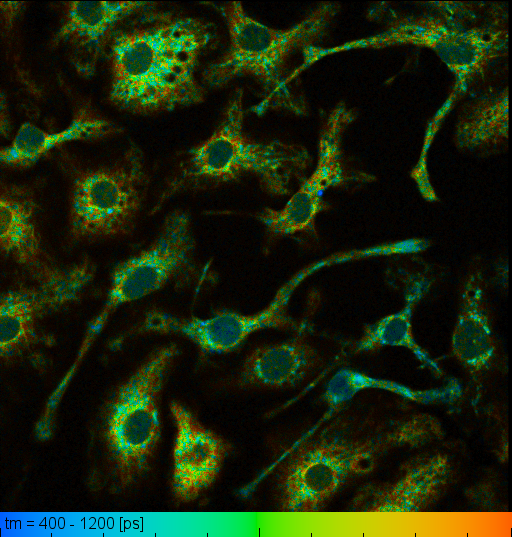

Supplement: Supplementary file 8 — Source data Fig. 6 [file 44319_2024_351_MOESM8_ESM.zip › 6H/BOTH 03_color_image.tif]

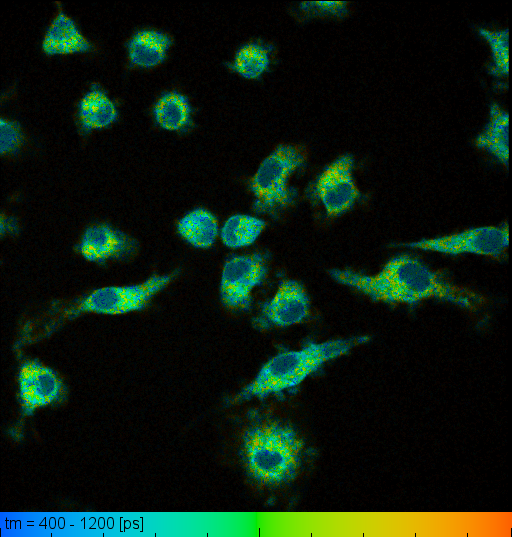

Supplement: Supplementary file 8 — Source data Fig. 6 [file 44319_2024_351_MOESM8_ESM.zip › 6H/CTRL 03_color_image.tif]

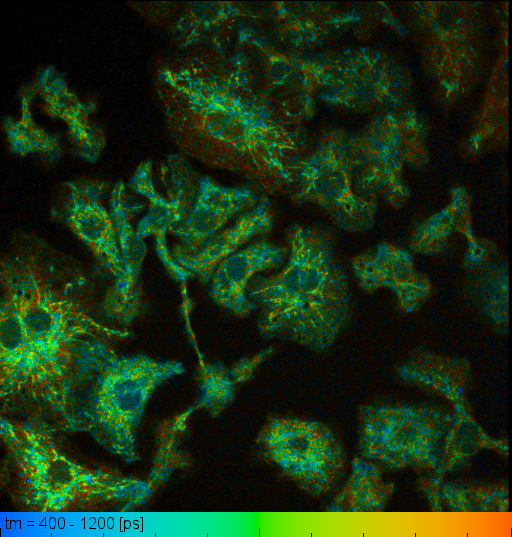

Supplement: Supplementary file 8 — Source data Fig. 6 [file 44319_2024_351_MOESM8_ESM.zip › 6H/P1C 03_color_image.tif]

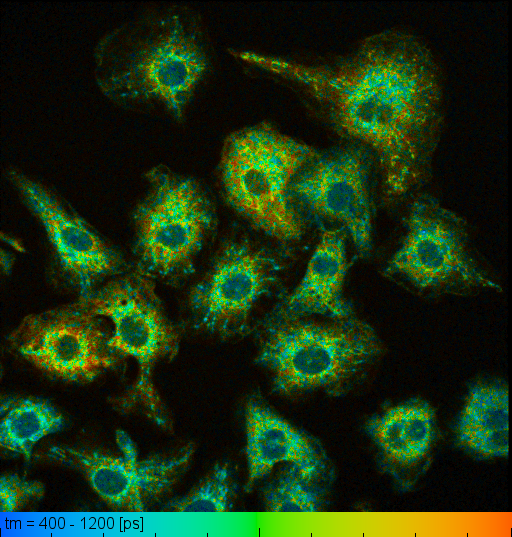

Supplement: Supplementary file 8 — Source data Fig. 6 [file 44319_2024_351_MOESM8_ESM.zip › 6H/PAM3 02_color_image.tif]

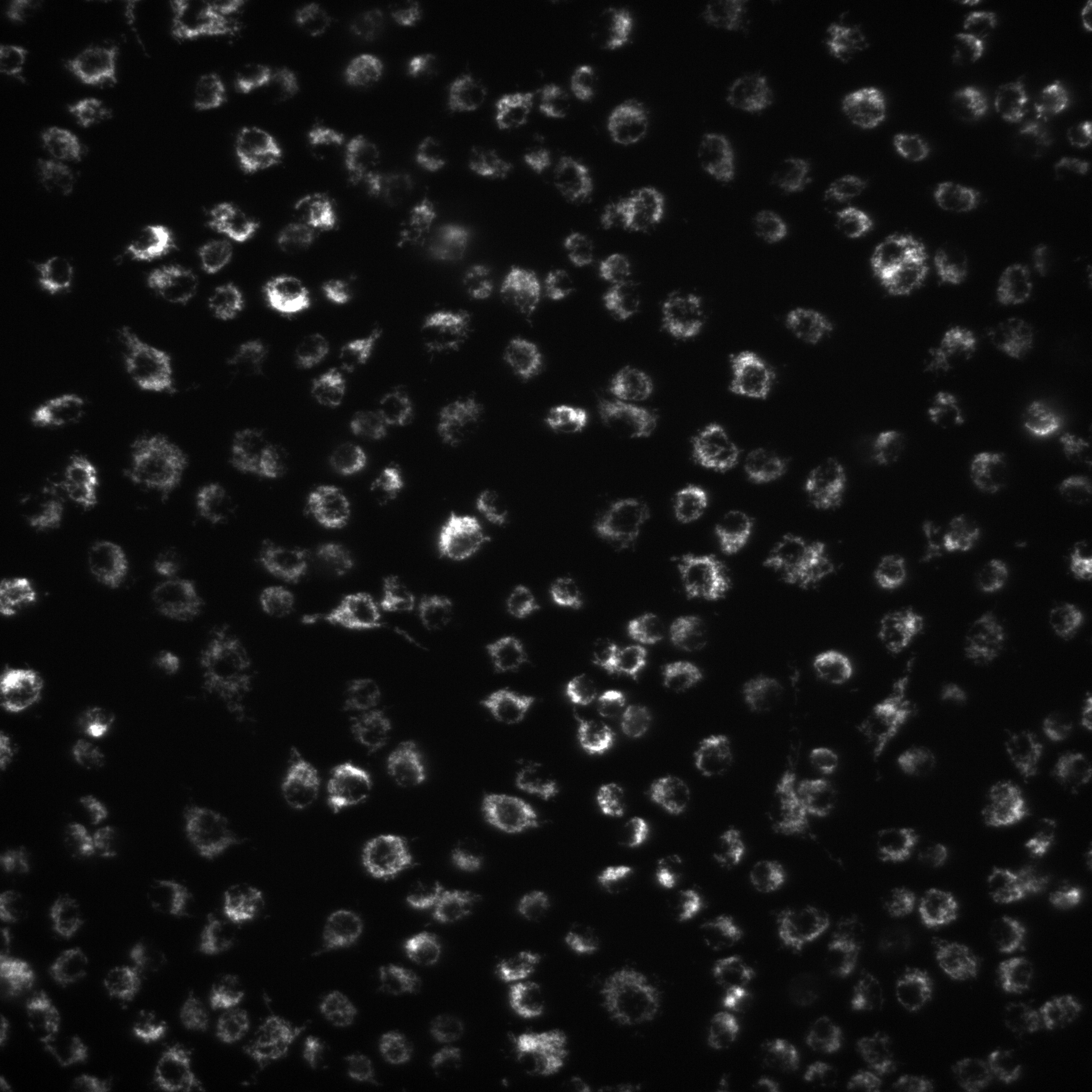

Supplement: Supplementary file 8 — Source data Fig. 6 [file 44319_2024_351_MOESM8_ESM.zip › 6F/B8.tif]

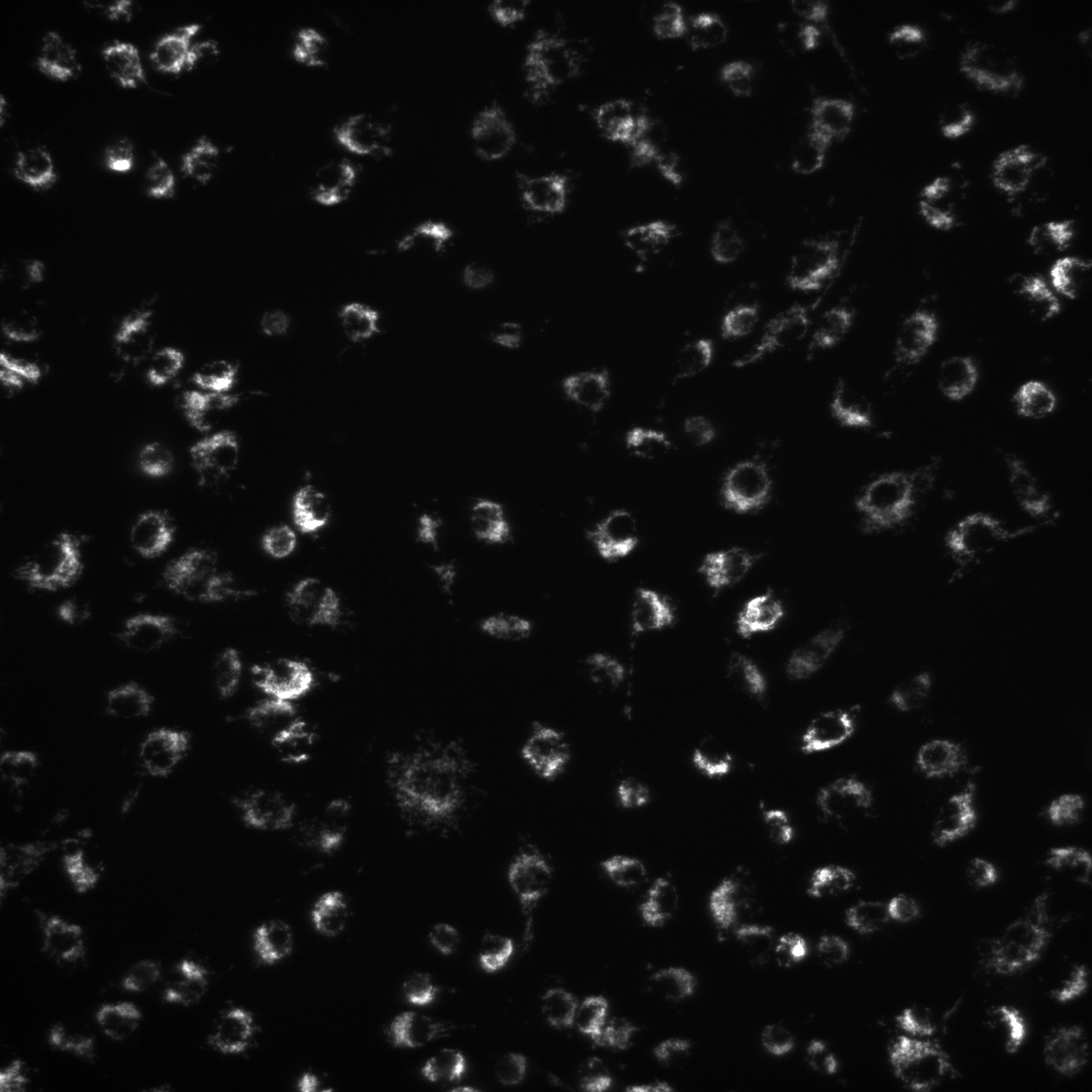

Supplement: Supplementary file 8 — Source data Fig. 6 [file 44319_2024_351_MOESM8_ESM.zip › 6F/C8.tif]

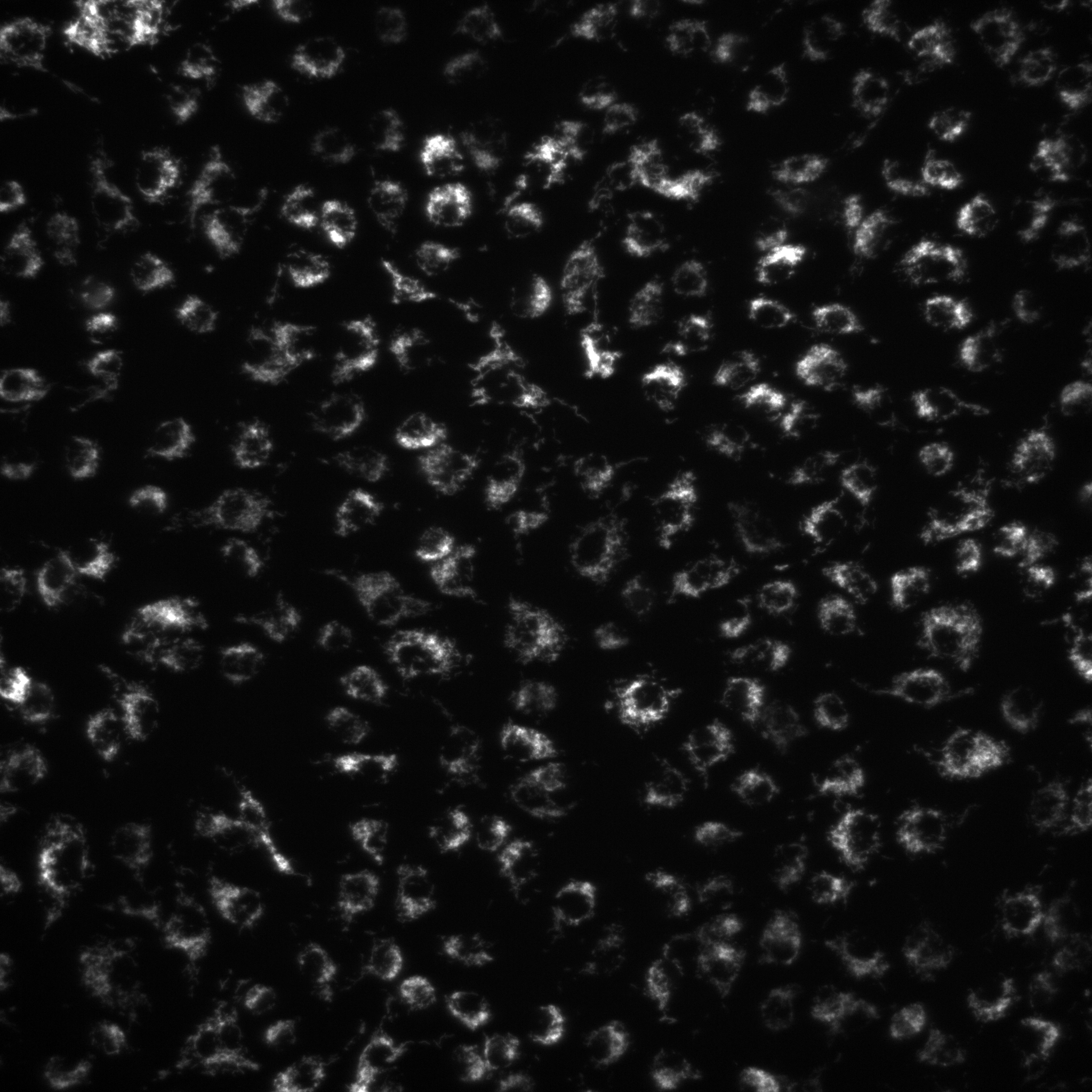

Supplement: Supplementary file 8 — Source data Fig. 6 [file 44319_2024_351_MOESM8_ESM.zip › 6F/D9.tif]

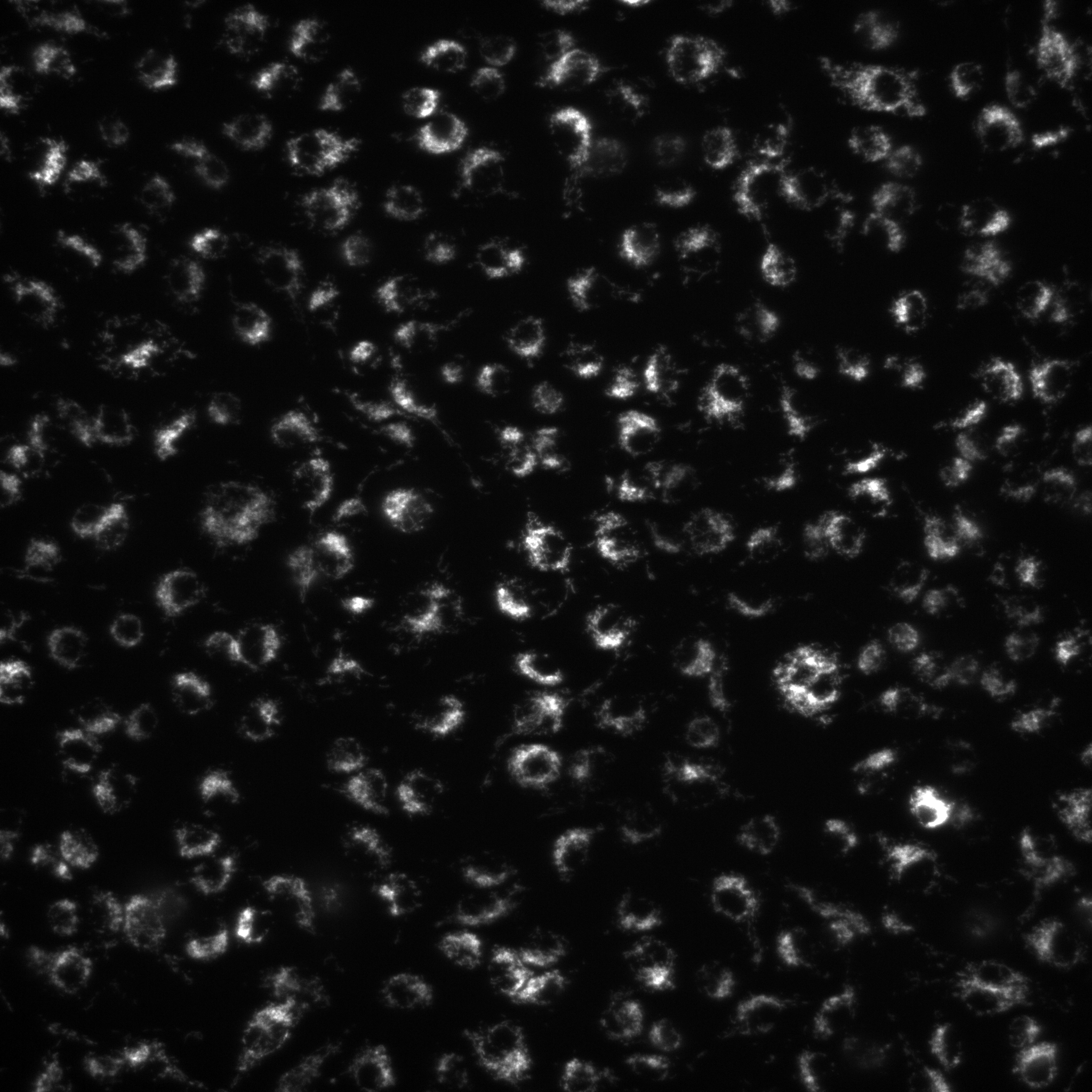

Supplement: Supplementary file 8 — Source data Fig. 6 [file 44319_2024_351_MOESM8_ESM.zip › 6F/E7.tif]
