# Supplementary material for: Pro-inflammatory macrophage activation does not require inhibition of oxidative phosphorylation
Source: EMBO Rep. 2025 Jan 3;26(4):982–1002. doi: 10.1038/s44319-024-00351-y (PMC11850891; doi:10.1038/s44319-024-00351-y)
Supplement: Supplementary file 4 — Source data Fig. 2 [file 44319_2024_351_MOESM4_ESM.zip › README FIG 2.rtf]

Figure 2 includes data measuring respiration of BMDMs isolated from Myd88 Trif, or Ifnar null mice treated with several different pro-inflammatory stimuli and combinations of stimuli. 
