# Supplementary material for: Pro-inflammatory macrophage activation does not require inhibition of oxidative phosphorylation
Source: EMBO Rep. 2025 Jan 3;26(4):982–1002. doi: 10.1038/s44319-024-00351-y (PMC11850891; doi:10.1038/s44319-024-00351-y)
Supplement: Supplementary file 5 — Source data Fig. 3 [file 44319_2024_351_MOESM5_ESM.zip › README FIG 3.rtf]

Figure 3 includes data from BMDMs and human PBMC-derived macrophages treated with pro-inflammatory stimuli for 24 hours. The measurements include metabolite accumulation and respiration. Additionally respiration of BMDMs isolated from mice lacking Irg1 is included. 
