# Supplementary material for: Pro-inflammatory macrophage activation does not require inhibition of oxidative phosphorylation
Source: EMBO Rep. 2025 Jan 3;26(4):982–1002. doi: 10.1038/s44319-024-00351-y (PMC11850891; doi:10.1038/s44319-024-00351-y)
Supplement: Supplementary file 6 — Source data Fig. 4 [file 44319_2024_351_MOESM6_ESM.zip › README FIG 4.rtf]

Figure 4 includes data from BMDMs treated with multiple combinations of pro-inflammatory stimuli for 24 hours. Measurements include permeabilized seahorse respirometry and stable isotope tracing from uniformly labeled 13C glucose or 13C glutamine. 
