# Supplementary material for: Pro-inflammatory macrophage activation does not require inhibition of oxidative phosphorylation
Source: EMBO Rep. 2025 Jan 3;26(4):982–1002. doi: 10.1038/s44319-024-00351-y (PMC11850891; doi:10.1038/s44319-024-00351-y)
Supplement: Supplementary file 7 — Source data Fig. 5 [file 44319_2024_351_MOESM7_ESM.zip › README FIG 5.rtf]

Figure 5 includes data from BMDMs treated with multiple combinations of pro-inflammatory stimuli and mitochondrial effector compounds for 24 hours. Measurements include gene expression, cytokine levels, and phagocytosis. Additionally validation of a CRISPR mediated deletion of Ndufs4 is included along with pro-inflammatory gene expression. 
