# Supplementary material for: Pro-inflammatory macrophage activation does not require inhibition of oxidative phosphorylation
Source: EMBO Rep. 2025 Jan 3;26(4):982–1002. doi: 10.1038/s44319-024-00351-y (PMC11850891; doi:10.1038/s44319-024-00351-y)
Supplement: Supplementary file 8 — Source data Fig. 6 [file 44319_2024_351_MOESM8_ESM.zip › README FIG 6.rtf]

Figure 6 includes data from BMDMs treated with multiple combinations of pro-inflammatory stimuli and mitochondrial effector compounds for 4 hours. Measurements include a time course of pro-inflammatory gene expression, respiration, and nitric oxide production. Additionally multiple orthogonal measurements of mitochondrial bioenergetics are taken at 4 hours. Finally, metabolite accumulation, enrichment and permeabilized seahorse assays are included. 
