# Supplementary material for: Pro-inflammatory macrophage activation does not require inhibition of oxidative phosphorylation
Source: EMBO Rep. 2025 Jan 3;26(4):982–1002. doi: 10.1038/s44319-024-00351-y (PMC11850891; doi:10.1038/s44319-024-00351-y)
Supplement: Supplementary file 9 — Source data Fig. 7 [file 44319_2024_351_MOESM9_ESM.zip › README FIG 7.rtf]

Figure 7 includes data from peritoneal macrophages isolated from mice treated with PBS or LPS for 24 hours. Measurements include pro-inflammatory gene expression, cytokine levels, metabolite accumulation, lactate efflux, and respiration. 
